# *BcHK71* and *BcHK67*, Two-Component Histidine Kinases, Regulate Conidial Morphogenesis, Glycerol Synthesis, and Virulence in *Botrytis cinerea*

**DOI:** 10.3390/jof11120850

**Published:** 2025-11-29

**Authors:** Mengjing Wang, Shiyu Gu, Jian Guo, Jingyu Wu, Xinhe Wang, Muhammad Noman, Jiaoyu Wang, Ling Li

**Affiliations:** 1 Zhejiang Key Laboratory of Biology and Ecological Regulation of Crop Pathogens and Insects, College of Advanced Agricultural Sciences, Zhejiang A&F University, Hangzhou 311300, China; wangmengjing_543@163.com (M.W.); gushiyu125422@163.com (S.G.); 16634253674@163.com (J.W.); 15368240008@163.com (X.W.); 2 College of Food and Health (College of Modern Food Industry), Zhejiang A&F University, Hangzhou 311300, China; jguo@zafu.edu.cn; 3 State Key Laboratory for Managing Biotic and Chemical Treats to the Quality and Safety of Agro-Products, Institute of Plant Protection and Microbiology, Zhejiang Academy of Agricultural Sciences, Hangzhou 310021, China; m.noman@zju.edu.cn

**Keywords:** *BcHK71*, *BcHK67*, *Botrytis cinerea*, functional characterization, histidine kinase

## Abstract

Fungal two-component signaling systems comprise histidine kinases (HKs), phosphotransfer intermediates, and response regulators. HKs are classified into eleven groups based on domain architecture; however, Group XI members in *Botrytis cinerea* remain uncharacterized. In this study, we investigated the functions of two Group XI histidine kinase genes, *BcHK71* and *BcHK67*, in *B. cinerea* via gene replacement. Phenotypic analysis revealed that *BcHK71* and *BcHK67* regulate conidiation, infection structures formation, and glycerol synthesis. Notably, *BcHK71* maintained cell wall integrity. Both genes also modulated expression of high osmolarity glycerol mitogen-activated protein kinase (HOG-MARK) signaling pathway components (*BcYpd1*, *BcSkn7*, *BcBos4*), while *BcHK67* uniquely upregulated *BcBrrg1* and enhanced BcHog1 phosphorylation. Transcriptomics analysis further indicated that *BcHK71* and *BcHK67* participated in pathways related to carbohydrate and lipid transport, metabolism and secondary metabolite biosynthesis. Disruption of these processes reduced pathogenicity and altered fungicide sensitivity in *B. cinerea*, with the Δ*BcHK71* mutant exhibiting more severe pronounced defects. Collectively, our findings underscore the critical roles of *BcHK71* and *BcHK67* in fungal development and pathogenicity, highlighting their potential as novel targets for controlling fungal diseases and managing fungicide resistance.

## 1. Introduction

*Botrytis cinerea*, a necrotrophic fungal pathogen, infects more than 1000 plant species, including most fruits and vegetables, and causes significant global economic losses [1,2]. Ranked among the top ten most destructive plant pathogens worldwide [3], it produces germinating spores that develop two distinct infection structures (IFSs) on host surfaces: appressoria (AP) and multicellular infection cushion (IC) [4]. Appressoria form within hours to days after germination, whereas infection cushions typically develop 24–48 h later. Both structures adhere firmly to the plant surface, secrete cell wall-degrading enzymes to breach host tissues, and enable direct penetration [5,6].

The two-component signaling system is an evolutionarily conserved mechanism that allows prokaryotes and eukaryotes to sense and adapt to environmental stimuli. Signal transduction, mediated by protein interactions and phosphorylation, regulates critical cellular processes [7,8,9]. In most eukaryotes, it involves a multistep phosphorylation cascade consisting of three key molecules: histidine kinases (HKs), histidine-containing phosphotransfer (HPt), and response regulators (RRs) [10,11,12]. HKs contain an input domain, a kinase domain, and a C-terminal acceptor domain with a conserved aspartate residue, defining them as heterotrimeric proteins with a modular tripartite structure [10,13,14]. The HPt acts as a unique phosphotransfer intermediate that interacts with both HKs and RRs [15].

Upon perception of environmental signals, HKs undergo autophosphorylation at a conserved histidine residue located in their kinase domain. The phosphate group is then sequentially transferred: first to an aspartate in the C-terminal receiver domain of the same HKs, then to a histidine on a HPt, and ultimately to an aspartate within the receiver domain of the RRs. The multistep His-Asp-His-Asp phosphorelay mechanism activates the functional domains of RR, initiating downstream signaling [13,16,17]. In eukaryotic organisms, two-component systems often serve as initiators of more complex intracellular signaling networks that incorporate additional components such as mitogen-activated protein kinase (MAPK) cascades and cyclic nucleotides-mediated pathways [18]. For instance, in *Saccharomyces cerevisiae*, under hyperosmotic conditions, the transmembrane histidine kinase *Sln1* is dephosphorylated [19]. This kinase normally acts as a negative regulator of a downstream MAPK cascade via the phosphorelay intermediates Ypd1 and Ssk1. Inactivation of Sln1 relieves this inhibition, enabling the sequential activation of the MAPKKK (Ssk2/Ssk22), the MAPKK (Pbs2), and finally the MAPK Hog1 [19,20]. Once phosphorylated, Hog1 translocates into the nucleus, where it modulates transcription factors activity to drive the expression of genes required essential for osmotic adaptation [21].

In filamentous fungi, HKs sense diverse environmental signals to regulate morphology, spore production, cell wall integrity, and pathogenicity [22]. Interestingly, the repertoire of HKs genes repertoire varies considerably among substantially across species: *B. cinerea* encodes twenty HKs genes, *Neurospora crassa* eleven, *Magnaporthe oryzae* ten, and *Gibberella moniliformis* sixteen, whereas *S. cerevisiae* possesses only one (*SLN1*) [13,23,24]. Fungal HKs are classified into eleven phylogenetically distinct groups, though not all groups are universally conserved [25]. For instance, *N. crassa* lacks Groups II and VII and *Cochliobolus heterostrophus* lacks Group IV, while *S. cerevisiae* possesses only Group VI HKs gene [13]. Moreover, the functions of different groups—and even orthologs within the same group—vary across species. For example, deletion of *S. cerevisiae SLN1* result in constitutive activation of the high-osmolarity glycerol (HOG) signaling pathway, excessive glycerol accumulation, and cell death [19,26]. In contrast, deletion of the Group VI HKs gene *FgSln1* in *Fusarium graminearum* reduced mycelial branching and pathogenicity but did not affect deoxynivalenol (DON) production [27,28,29]. Group III HKs exhibit relatively conserved regulatory roles. The *N. crassa* gene *NcNIK-1*/*OS-1*, which encodes a Group III HKs, regulates osmotic adaptation, mycelial growth, and sensitivity to dicarboximide and phenylpyrrole fungicides [13]. Similarly, *B. cinerea BOS1* (a Group III ortholog) is critical for oxidative stress tolerance and pathogenicity [30].

In summary, although filamentous fungi may encode up to 11 types of HKs, research in *B. cinerea* has primarily focused on Groups III and VI. To broaden understanding of other HKs in this pathogen, we selected two Group XI HK genes, *BcHK71* and *BcHK67*, for functional characterization. This study aimed to elucidate their roles in growth, development, pathogenesis, and osmotic stress response. By investigating these HKs, we sought to provide deeper insights into the molecular mechanisms of *B. cinerea* pathogenicity and to identify potential targets for novel control strategies.

## 2. Materials and Methods

### 2.1. Fungal Strains, Culture Conditions, and Transformation

The wild-type (WT) *B. cinerea* strain B05.10, along with all gene knockout mutants (Δ*BcHK71*-A, Δ*BcHK71*-B, Δ*BcHK67*-A, Δ*BcHK67*-B), and an ectopic transformant (ET) carrying a random insertion of the transformation vector and serves as a control, were maintained at 22 °C on solid complete medium (CM) [31]. The CM contained per liter: 10 g of D-glucose as carbon source, 2 g of peptone, 1 g of yeast extract, 1 g of casamino acids, nitrate salts (6 g NaNO_3_, 0.52 g MgSO_4_⋅7H_2_O, 1.52 g KH_2_PO_4_), trace elements, 0.1 g vitamin mix (biotin, pyridoxine, thiamine, riboflavin, *p*-aminobenzoic acid, and nicotinic acid). The pH was adjusted to 6.5 prior to sterilization. All fungal transformants were generated using *Agrobacterium tumefaciens*-mediated transformation (*At*MT) [32]. Primary transformants were selected on CM plates supplemented with 250 µg/mL hygromycin B (Roche, Mannheim, Germany; Cat. No. 10843555001) and incubated at 22 °C. For dry weight measurements, mycelia from shake cultures (22 °C, 120 rpm, 5 d) in liquid CM were harvested by vacuum filtration, washed thoroughly with distilled water, and dried to a constant weight at 60 °C before weighing.

### 2.2. Nucleic Acid Extraction and cDNA Synthesis

Genomic DNA and total RNA were extracted from fresh mycelia of WT *B. cinerea* B05.10, gene deletion mutants (Δ*BcHK71*-A/B and Δ*BcHK67*-A/B), and the ET strain. Mycelia were grown statically for 5 d at 22 °C in the dark in 6-well plates containing liquid CM. DNA was isolated using Sangon Biotech’s Fungal DNA Isolation Kit (Cat# B518629) according to the manufacturer’s instructions. The concentration and purity of the DNA were determined using a NanoDrop 2000 spectrophotometer (Thermo Fisher Scientific, Waltham, MA, USA). Total RNA was isolated using Sangon Biotech’s Fungal RNA Isolation Kit (Cat# B518229). The quality of the extracted RNA was assessed by measuring the A260/A280 ratio with the NanoDrop 2000, and only samples with a ratio between 1.8 and 2.0 were used for subsequent experiments. Subsequently, cDNA was synthesized from 1 µg of total RNA (with an A260/A280 ratio between 1.8 and 2.0) using the HiScript Ⅲ All-in-one RT SuperMix Perfect for qPCR Kit (Vazyme, Cat# R333-01). This kit features an integrated genomic DNA removal step, where contaminating genomic DNA is efficiently digested by a heat-labile DNase I during a dedicated incubation step prior to the reverse transcription reaction, thereby ensuring the purity of the cDNA for subsequent qPCR analysis.

### 2.3. Bioinformatic Analysis

Homologs of BcHK71 and BcHK67 in *B. cinerea* were identified using the NCBI BLAST. Coding sequences were amplified from cDNA with primers bcHK71-innerf1/bcHK71-innerr1 and bcHK67-innerf1/bcHK67-innerr1 and confirmed by bidirectional Sanger sequencing (HangZhou Youkang Biology Co., Ltd., Hangzhou, China) (Table S1). Homologous proteins from other species were retrieved via NCBI BLASTp using BcHK71 and BcHK67 as queries (Table S2). Multiple sequence alignment were generated with MUSCLE in MEGA11 [33], manually refined in GENEDOC [34], and subsequently used to construct phylogenetic trees in MEGA11 with the Neighbor-Joining method under the minimum evolution model [33,35]. Bootstrap support values were calculated from 1000 replicates. Protein domains were predicted using Pfam (http://pfam-legacy.xfam.org/, accessed on 15 March 2023) [36] and SMART (https://smart.embl.de/, accessed on 19 March 2023) [37] and visualized with Domain Graph DOG software (v1.0) [38].

### 2.4. Gene Deletion and Mutant Verification

Upstream flanking fragments (1.5 kb, *Sal* I/*Hind* III) and downstream (*EcoR* I/*Xho* I) flanking fragments of *BcHK71* and *BcHK67* were amplified from genomic DNA of the WT strain B05.10 using specific primers provided in Table S1. The purified flanking fragments and the linearized p1300-KO vector were assembled with the ClonExpress Ultra One Step Cloning Kit V2 (Vazyme, Nanjing, China; Cat, No. C116-02) according to the manufacturer’s instructions to generate the knockout vectors Pko-BcHK71 and Pko-BcHK67 [39]. These vectors were introduced into *A. tumefaciens* strain *AGL 1* by electroporation. Fungal transformation was then performed via *At*MT as previously described, with co-cultivation on induction medium for *A. tumefaciens* (IMAS) plates at 22 °C for 48–72 h [32]. IMAS medium consisted of induction medium (IM) supplemented with 200 µM acetosyringone (AS) [32]. Hygromycin B-resistant strains were screened by PCR. Two types of strains were isolated and characterized: (1) Targeted gene knockout mutants, generated by homologous replacement of the target gene with the *hph* cassette. (2) ET control strain, resulting from random, non-homologous integration of the entire T-DNA elsewhere in the genome without disrupting the target gene. ET strains served as essential controls for the presence of T-DNA and the hygromycin resistance gene.

Both Δ*BcHK71* mutants and ET control strains were verified using PCR strategy with the following primer sets: BcHK71-innerF/BcHK71-innerR (1595 bp target), BcHK71-UP/HPH-UP (amplifying the 1785 bp upstream junction), BcHK71-DN/HPH-DN (1797 bp downstream junction), and HPH-F/HPH-R (to amplify a 1001 bp *hph* fragment). Similarly, Δ*BcHK67* mutants were verified with BcHK67-innerF/BcHK67-innerR (1552 bp target), BcHK67-UP/HPH-UP (1803 bp upstream junction), BcHK67-DN/HPH-DN (1759 bp downstream junction), and HPH-F/HPH-R (amplifying the 1001 bp *hph*). RT-qPCR was performed with HK71RTF/HK71RTR for *BcHK71* and HK67RTF/HK67RTR for *BcHK67*, using *UCE* (BC1G_14594) as the internal control. Each 20 µL reaction contained 10 µL SYBR Green Master Mix (Vazyme, Nanjing, China, Cat. No. Q311-02), 0.8 µL each primer (10 µM), 2 µL cDNA (diluted 1:10), and 6.4 µL nuclease-free water. Each sample was run in triplicate (technical replicates) on a CFX Connect Real-Time System (BIO-RAD, Singapore). The amplification protocol consisted of an initial denaturation step at 95 °C for 10 min, followed by 40 cycles of denaturation at 95 °C for 15 s and annealing/extension at 60 °C for 1 min. Gene expression levels were quantified using the comparative Ct (2^–ΔΔCt^) method [40], with results normalized to the WT control. Three biological replicates were performed. Two independent mutant strains for each gene were selected for all subsequent phenotypic analyses to ensure reproducibility.

### 2.5. Fungal Developmental Assays

Mycelial growth, conidiation, conidial morphology, conidial germination, AP, and IC formation were assessed as described previously [41]. For the mycelial growth assay, 5 mm pieces of the edge mycelium from each strain grown for 3 d were inoculated onto CM and incubated at 22 °C in the dark for 2–8 d. Photographs were taken, and the diameter of the mycelium was measured on day 2. For conidiation assays, conidia from 10 d-old CM cultures were harvested by washing with 4 mL sterile distilled water, filtered, resuspended in 2 mL of water, and counted using a hemocytometer under a microscope. Conidial germination was assessed by incubating 20 µL of conidial suspension (1 × 10^5^ spores/mL in 10 mM fructose) on coverslips (Fisher, Waltham, MA, USA; Cat. No 22261936) at 22 °C in the dark, with germination rates recorded at 2, 4, 6, 8, 10, 12, 24, and 48 h post-inoculation (hpi). For IFS formation, a 2 µL drop of conidial suspension (5 × 10^5^ spores/mL) was mixed with 20 µL liquid CM on a slide and incubated under the same conditions. AP formation was monitored at 6, 8, 10, 12, 24, and 48 hpi following conidial germination, while IC were specifically examined at 24 and 48 hpi, corresponding to the established developmental period for mature IC formation in *B. cinerea* [5]. For IC formation on onion epidermis, 20 µL conidial suspension (1 × 10^5^ spores/mL in 10 mM fructose) was inoculated onto the inner epidermis and incubated for 48 h at 22 °C. Samples were stained with cotton blue-lactophenol for 5 min, rinsed with sterile distilled water, and examined microscopically [42]. All experiments were performed with three independent biological replicates.

### 2.6. Pathogenicity Tests

Pathogenicity assays were conducted on intact tomato leaves, wounded apple fruits, tomato fruits, and strawberry fruits. Plant materials were selected based on developmental stage and overall health, surface-sterilized with 1% NaClO for 3 min followed by 70% ethanol for 30 s and then air-dried. Fruits were wounded to depth of 3 mm with a sterile needle. Mycelia plugs (5 mm) from 3 d CM cultures of WT, mutants, and ET were inoculated. Samples were incubated at 25 °C under a 12 h light/12 h dark cycle. Photographs were taken on day 3, and lesion diameters were measured using ImageJ software (v1.53e) [43]. Each treatment included three biological replicates with three technical replicates per host. WT and mock-inoculated (CM-only) plugs served as controls.

### 2.7. Stress Adaptation Assays

Mycelial plugs (5 mm in diameter) taken from 3 d-old CM cultures were inoculated on CM agar supplemented with cell wall disrupting agents (0.3 mg/mL Congo Red and 0.05 mg/mL Calcofluor White), or with fludioxonil (0.00625 μg/mL), and incubated in the dark at 22 °C for 3 d. After incubation, the colonies were photographed, and the radial growth was measured.

For glycerol content determination, mycelial plugs of the WT, Δ*BcHK71*-A, Δ*BcHK71*-B, Δ*BcHK67*-A, Δ*BcHK67*-B, and ET strains were inoculated into YEPD liquid medium [44] and cultured at 22 °C with shaking at 150 rpm for 48 h to compare the basal glycerol levels among genotypes. Glycerol content was determined using a Tissue Glycerol Assay Kit (Applygen Technologies Inc., Beijing, China, Cat. No. E1012), according to the manufacturer’s instructions with minor modifications. Briefly, after cultivation, mycelial samples (~100 mg; exact weight recorded for normalization) were homogenized in 1 mL of lysis buffer using an electric homogenizer and incubated for 10 min. The homogenate was centrifuged at 5000× *g* for 10 min, and the supernatant was heated at 70 °C for 10 min to inactivate endogenous enzymes. After a second centrifugation, the supernatant was collected as the test sample. Absorbance was measured at 550 nm using a microplate reader. A standard curve was generated with glycerol standards provided in the kit, and the glycerol concentration in the samples was calculated based on the standard curve. The glycerol content was quantified using a standard curve (y = 0.0004x + 0.0185, R^2^ = 0.9917) and normalized to micromoles per gram of fresh weight (μmol/g FW).

For abiotic stress, strains were grown on CM with osmotic stressors (1 M NaCl, 1 M KCl, 1 M sorbitol, and 1 M glycerol), oxidative stressors (2 mM methyl viologen, 5 mM H_2_O_2_), membrane stress (0.005% sodium dodecyl sulfate, SDS), or ionic stress (0.5 M CaCl_2_). Growth was measured relative to controls. All experiments were performed with three independent biological replicates.

### 2.8. Western-Blot Analysis of Phosphorylation of Hog1

Mycelia from 2 d-old liquid cultures were collected and mechanically pulverized in liquid nitrogen. The powdered tissue was subsequently homogenized in protein extraction buffer containing 62.5 mM Tris-HCl (pH 6.8), 10% glycerol, 2% SDS, 50 mM dithiothreitol, 1 mM phenylmethylsulfonyl fluoride (PMSF), 10 mM β-mercaptoethanol, and a protease inhibitor cocktail (Sigma-Aldrich, St. Louis, MO, USA). After centrifugation at 12,000× *g* for 10 min at 4 °C, the supernatant proteins were separated by 10% SDS-PAGE gels and electrotransferred onto an Immobilon-P transfer membrane (Millipore, Burlington, MA, USA). Phosphorylated Hog1 was detected using a rabbit monoclonal anti-Phospho-p38 MAPK (Thr180/Tyr182) (D3F9) XP^®^ antibody (Cell Signaling Technology, Beverly, MA, USA; Cat. No. 8632S), followed by incubation with a rabbit polyclonal anti-GAPDH antibody (HuaBio, Woburn, MA, USA; Cat. No. R1208-3; 1:5000 dilution) as the loading control. Band intensities were quantified using ImageJ software [42]. Background subtraction was applied, and the relative phosphorylation level of Hog1 was calculated by normalizing the phospho-Hog1 band intensity to that of the corresponding GAPDH band. All experiments were repeated three times.

### 2.9. Ecto-ATPase Activity Assay

(Ca^2+^-Mg^2+^)-ATPase was assayed using a commercial kit (Sangon Biotech, Shanghai, China; Cat# D799644) by quantifying inorganic phosphate (Pi) liberation. Fresh mycelia (0.1 g) were homogenized in 1 mL of ice-cold Reagent 1 and centrifuged at 8000× *g* for 10 min at 4 °C. The supernatant was used for immediate analysis. The assay was performed according to the manufacturer’s instructions by incubating 200 μL of supernatant with 90 μL Reagent 1, 80 μL Reagent 2, and 40 μL Reagent 3 at 25 °C for 10 min. Parallel control tubes received sample after reaction termination. Reactions were stopped with 50 μL Reagent 5, then centrifuged at 4000× *g* for 10 min. Liberated Pi in 100 μL supernatant was measured at 660 nm after reaction with freshly prepared phosphomolybdate reagent. Activity was calculated as per the kit’s calculation instructions:

ATPase activity (U/g) = 7.5 × (ΔA_sample_/ΔA_standard_)/W

where ΔA_sample_ = A_test_ − A_control_, ΔA_standard_ = A_standard_ − A_blank_, and W = sample mass (g). One unit liberates 1 μmol Pi per hour per gram fresh tissue.

### 2.10. Transcriptome Analysis

Mycelia from WT strain B05.10 and deletion mutants (Δ*BcHK71* and Δ*BcHK67*) grown in liquid CM (180 rpm, 48 h) were collected, stored in liquid nitrogen, and sent to Biomarker Technologies (Beijing, China) for RNA sequencing. Total RNA was extracted using TRIzol Reagent (Invitrogen, Waltham, MA, USA). RNA quality (RIN) > 8.0 was checked with Agilent 2100 /LabChip GX (Agilent Technologies, Santa Clara, CA, USA) and a NanoDrop 2000 spectrophotometer. Following the enrichment of poly(A) mRNA from total RNA, sequencing libraries were constructed using the NEBNext Ultra II RNA Library Prep Kit for Illumina (New England Biolabs, Ipswich, MA, USA). Libraries were prepared with NEBNext Ultra II RNA Library Prep Kit and sequenced on Illumina platform with poly(A) mRNA enrichment. The libraries were sequenced on an Illumina NovaSeq 6000 (150 bp paired-end reads, ~40 million reads/sample). Clean reads were filtered from raw sequencing data using Cutadapt (v1.16) to remove low-quality sequences and stored in FASTQ format [45]. These clean reads were aligned to the *B. cinerea* B05.10 reference genome (NCBI) using HISAT2 (v2.1.0) [46]. Gene expression levels were estimated as FPKM (fragments per kilobase of transcript per million mapped reads) using StringTie (v1.3.3b) [47]. Differentially expressed genes (DEGs) were identified using DESeq2 (|log_2_ fold change| ≥5, FDR <0.001) [48]. Functional enrichment analyses of Kyoto Encyclopedia of Genes and Genomes (KEGG) pathways [49] and Gene Ontology (GO) terms [50] were conducted on the BMKCloud platform (https://www.biocloud.net/, accessed on 26 May 2024). The complete dataset generated from the GO enrichment analysis is provided as Supplementary Dataset S1 (Excel file). Heat map visualization was generated with pheatmap (v1.0.12) for hierarchical clustering [51] and the ggplot2 package (V3.4.0) for graphical refinement [52].

### 2.11. RT-qPCR Analysis of the Botrydial Biosynthetic Gene Cluster and HOG-MAPK Pathway Genes

RT-qPCR was performed to verify the expression of differentially expressed genes from the botrydial biosynthetic gene cluster and selected genes from the HOG-MAPK pathway, using the primer pairs listed in Supplementary Table S1. These primers were designed using Primer5.0 software [53]. The reference gene *UCE* (BC1G_14594) was used as an internal control. Each 20 µL reaction contained 10 µL SYBR Green Master Mix (Vazyme, Nanjing, China; Cat# Q311-02), 0.8 µL of each primer (10 µM), 2 µL cDNA (diluted 1:10), and 6.4 µL nuclease-free water. Each sample was run in triplicate (technical replicates) on a CFX Connect Real-Time System (BIO-RAD, Singapore). The amplification protocol consisted of an initial denaturation step at 95 °C for 10 min, followed by 40 cycles of denaturation at 95 °C for 15 s and annealing/extension at 60 °C for 1 min. Gene expression levels were quantified using the comparative Ct (2^−ΔΔCt^) method [40], with results normalized to the WT control. Three biological replicates were performed.

### 2.12. Microscopy and Image Analyses

The conidial morphology, conidiophore clusters, AP and IC were observed with an OLYMPUS CX33 microscope. Images were captured with ImageView software (v4.10) [54]. Colony morphology, sclerotia, and lesions were photographed with a Nikon camera. Images were processed with Adobe Photoshop 2020.

### 2.13. Statistical Analysis

All quantitative data are presented as the mean ± standard deviation (SD) from three independent biological replicates with three technical replicates each. Differences were assessed with Dunnett’s test in DPS software (v9.01) [55,56]. Datasets with *p* < 0.05 (*) or *p* < 0.01 (**) were considered significantly different from each other.

## 3. Results

### 3.1. Identification of BcHK71 and BcHK67 Genes in B. cinerea

**Figure 1:**
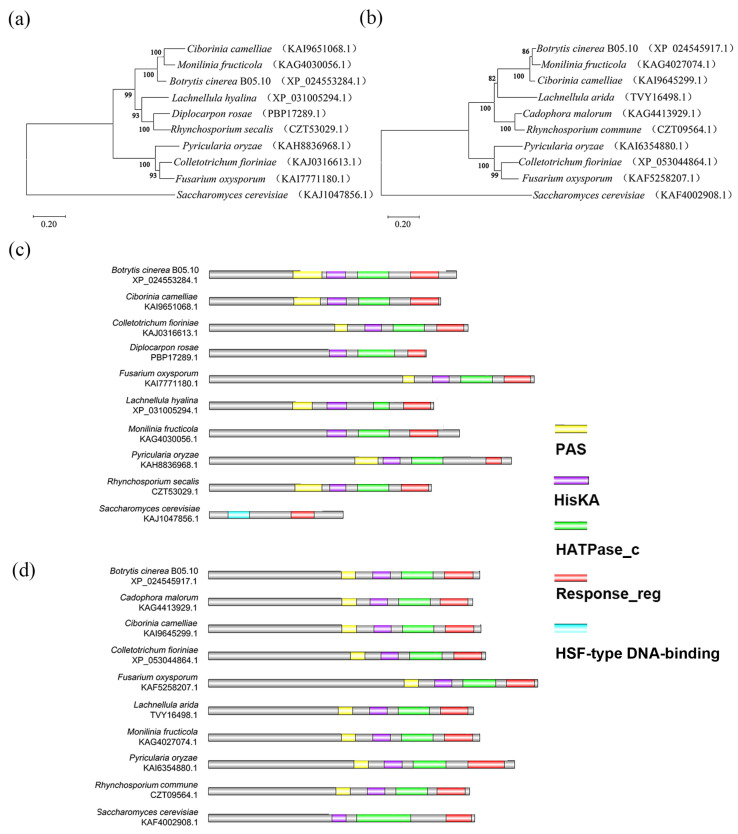
Phylogenetic and domain architecture analysis of BcHK71 and BcHK67. (**a**,**b**) Comparative phylogenetic analysis of BcHK71 (**a**) and BcHK67 (**b**) and their homologs, constructed using the neighbor-joining method in MEGA-X. The numbers at the branches represent bootstrap support values (%) from 1000 replicates. (**c**,**d**) Schematic representation of the conserved domain architectures of BcHK71 (**c**) and BcHK67 (**d**). Domain abbreviations: PAS, Per-ARNT-Sim (signal-sensing domain); HisKA, Histidine Kinase A (phosphoacceptor domain); HATPase_c, Histidine kinase-like ATPase (catalytic domain); Response_reg, Response regulator (phosphoreceiver domain).

*BcHK71* (BCIN_15g02150) and *BcHK67* (BCIN_01g00280) were identified as Group XI HKs in *B. cinerea* B05.10 genome (NCBI Assembly ASM83294v1; GenBank Accession GCA_000143535.3) through BLAST searches using orthologs described in reference [13]. Their coding sequences were verified via cDNA amplification and showed 100% identity with the *B. cinerea* B05.10 reference genome (Assembly ASM83294v1). The *BcHK71* gene contains a 3843 bp open reading frame (ORF) comprising four exons and three introns, and it encodes a predicted protein of 1148-amino acids residues. The predicted BcHK71 protein shares 70.93% and 69.41% identity with its orthologs from *Monilinia fructicola* (KAG4030056.1) and *Ciborinia camelliae* (KAI9651068.1), respectively (Figure S1). *BcHK67* contains a 3850 bp ORF with three exons and two introns, encoding 1244-amino acid residues, and shares 86.74% and 81.75% identity with orthologs from *M. fructicola* (KAG4027074.1) and *C. camelliae* (KAI9645299.1), respectively (Figure S2). A phylogenetic tree was constructed using the neighbor-joining method in MEGA-X to infer the phylogenetic relationships among sclerotiniaceous fungi. Both *BcHK71* and *BcHK67* clustered closely with orthologs from *C. camelliae* and *M. fructicola* (Figure 1a,b). Phylogenetic analysis showed that both BcHK71 and BcHK67 clustered closely with their orthologs from *C. camelliae* and *M. fructicola*, forming distinct clades (Figure 1a,b). SMART domain analysis predicted that both proteins contain Period-ARNT-Single-minded (PAS), histidine kinase A (HisKA), histidine kinase-like ATPase (HATPase_c), and response regulator (Response_reg) domains (Figure 1c,d).

### 3.2. Disruption of BcHK71 and BcHK67

**Figure 2:**
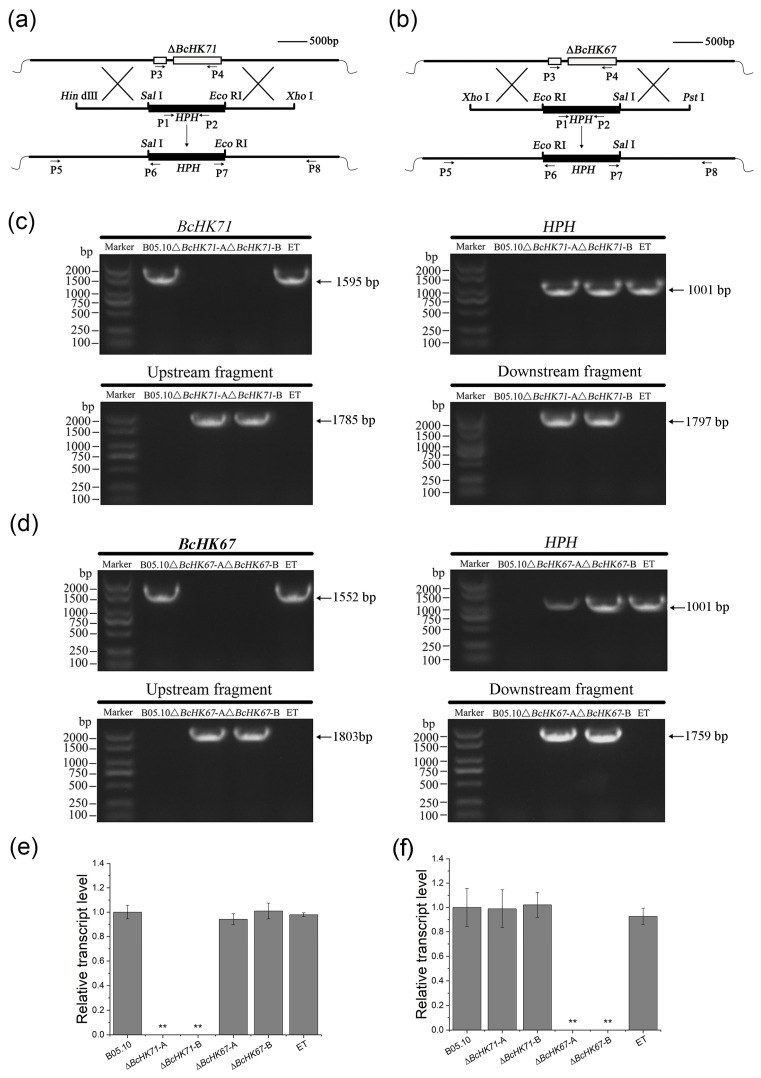
Generation and validation of *BcHK71* and *BcHK67* mutants in *Botrytis cinerea*. (**a**,**b**) Schematic diagrams illustrating the construction of the *BcHK71* (**a**) and *BcHK67* (**b**) gene knockout vectors via homologous recombination. The hygromycin B phosphotransferase (*hph*) gene replaces the target open reading frame. (**c**) PCR validation of *BcHK71* deletion. Expected amplicon sizes: 1595 bp (native *BcHK71*), 1001 bp (*hph* cassette), 1785 bp (upstream flank), and 1797 bp (downstream flank). (**d**) PCR validation of *BcHK67* deletion. Expected amplicon sizes: 1552 bp (native *BcHK67*), 1001 bp (*hph* cassette), 1803 bp (upstream flank), and 1759 bp (downstream flank). (**e**,**f**) Quantitative RT-PCR analysis of *BcHK71* in WT Δ*BcHK71* (**e**) and Δ*BcHKI67* (**f**) transcript levels in the WT, mutants (Δ*BcHK71*, Δ*BcHK67*), and ET strains. Values are mean ± SD from three independent experiments. ** *p* < 0.01 vs. WT (Dunnett’s test).

To generate deletion mutants of *BcHK71* and *BcHK67* in *B. cinerea* strain B05.10, homologous recombination was performed using the knockout vectors pKO-BcHK71 and pKO-BcHK67, respectively (Figure 2a,b). Hygromycin B-resistant transformants were selected and verified by PCR. Mutants showed amplification of the *hph* cassette (1001 bp) and the expected upstream/downstream junction fragments (*BcHK71*: 1785/1797 bp; *BcHK67*: 1803/1759 bp), but not the target genes ORFs (*BcHK71*: 1595 bp; *BcHK67*: 1552 bp) (Figure 2c,d). The WT strain amplified only the target gene, whereas the ET showed amplification of both the target gene and the *hph* cassette. Gene deletion was further confirmed by qRT-PCR: *BcHK71* expression was abolished in Δ*BcHK71* mutants but remained unchanged in Δ*BcHK67* and ET, while *BcHK67* expression was eliminated in Δ*BcHK67* mutants but unaffected in Δ*BcHK71* and ET (Figure 2e,f). Collectively, these results confirm the successful deletion of the target genes, namely *BcHK67* and *BcHK71*.

### 3.3. BcHK71 and BcHK67 Are Required for Vegetative Development

**Figure 3:**
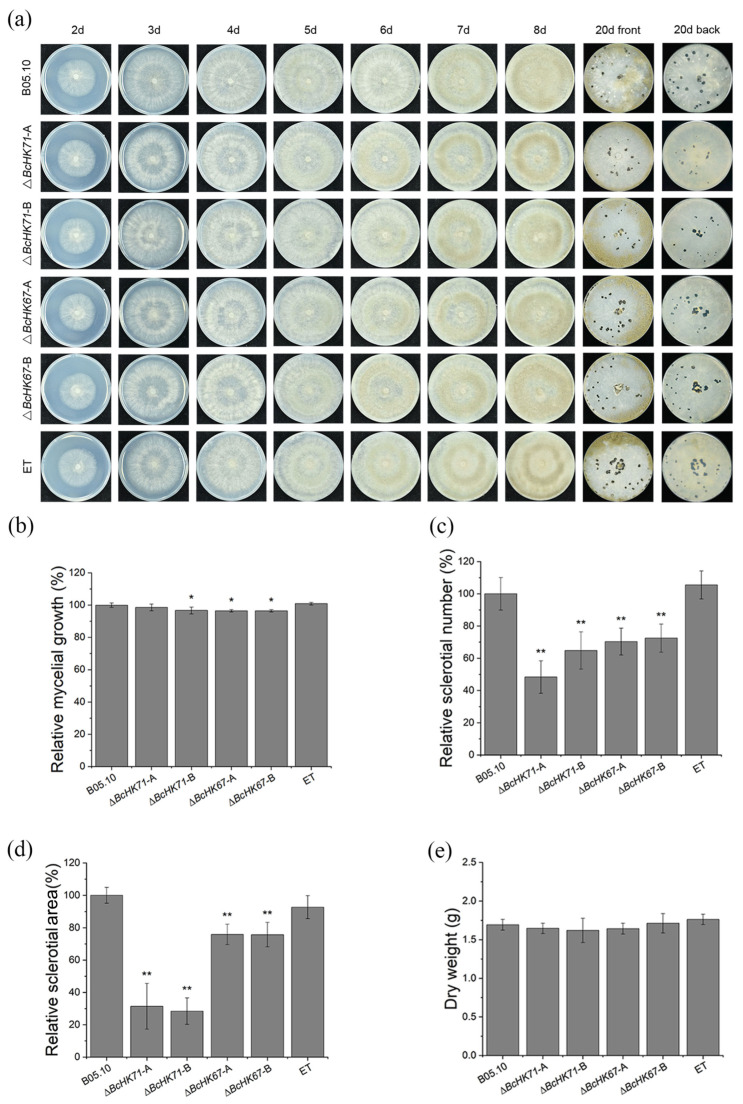
Deletion of *BcHK71* or *BcHK67* impairs vegetative growth and sclerotial development. (**a**) Colony morphology of the WT, mutant (Δ*BcHK71*-A, Δ*BcHK71*-B, Δ*BcHK67*-A, Δ*BcHK67*-B), and ET strains on CM at 2 to 8 days. Sclerotia formation was photographed at 20 days. (**b**) Colony diameters at 2 days. (**c**,**d**) Statistical analysis of sclerotia number (**c**) and area (**d**) per colony at 20 days. (**e**) Mycelial dry weight determined after 5 days of growth in liquid CM. Data represent the mean ± SD (n = 3). * *p* < 0.05, ** *p* < 0.01 vs. WT (Dunnett’s test).

Mycelial growth and sclerotia formation of WT, Δ*BcHK71*, Δ*BcHK67*, and ET strains were compared on CM. After two days, mutant colonies were slightly smaller than those of the WT and ET strains (Figure 3a,b). Both independent Δ*BcHK71* mutants produced fewer and smaller sclerotia than the WT and ET strains, showing a consistent trend despite minor variations in the degree of reduction (Figure 3a,c,d). No significant difference in mycelial dry weight was observed (Figure 3e). These results indicate that *BcHK71* and *BcHK67* are required for normal mycelial growth and sclerotia formation in *B. cinerea*.

### 3.4. BcHK71 and BcHK67 Are Involved in Asexual and IFSs Development

**Figure 4:**
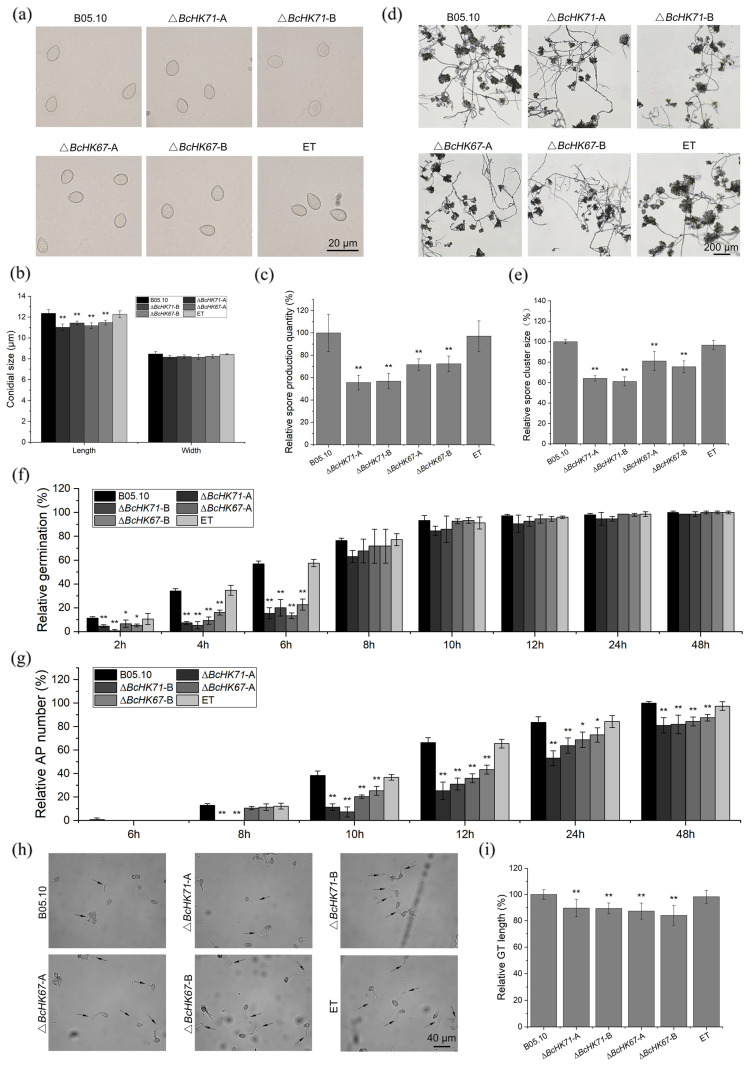
Deletion of *BcHK71* and *BcHK67* impairs conidiation and early infection-related development. (**a**) Morphology of conidia from the indicated strains. Scale bar = 20 µm. (**b**) Quantification of conidial length and width. (**c**) Conidial production after 10 days of culture on CM plates. (**d**,**e**) Morphology (**d**) and size quantification (**e**) of conidiophore clusters. (**f**,**g**) Time-course analysis of conidial germination rates (**f**) and appressorium (AP) formation rates (**g**) post-inoculation. (**h**,**i**) Representative images (**h**) and quantification (**i**) of germ tube (GT) length at 9 hpi. Scale bar in (**h**) = 40 µm. All quantitative data are presented as the mean ± standard deviation (SD) from three independent experiments (n = 3). Asterisks denote significant differences compared to the wild-type (WT) strain: * *p* < 0.05, ** *p* < 0.01 (Dunnett’s test).

**Figure 5:**
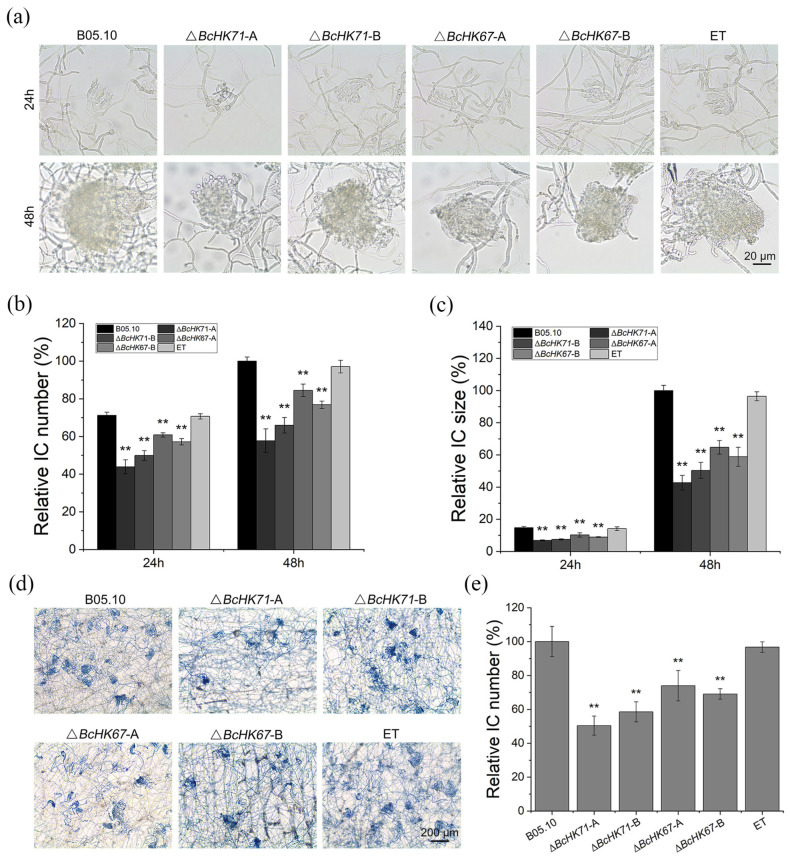
*BcHK71* and *BcHK67* are essential for infection cushion (IC) development. (**a**) Representative micrographs of ICs formed by the indicated strains on a hydrophobic surface at 24 and 48 hpi. (**b**,**c**) Quantitative analysis IC numbers (**b**) and size (**c**) per microscopic field on inductive surfaces. (**d**) IC formation on onion epidermis at 48 hpi. (**e**) Quantification of ICs formed on onion epidermis. All data are presented as the mean ± standard deviation (SD) from three independent biological replicates (n = 3). ** *p* < 0.01 vs. WT (Dunnett’s test).

To investigate the role of *BcHK71* and *BcHK67* in asexual reproduction, we examined conidial production, conidial morphology, and conidiophore architecture. Conidia from the Δ*BcHK71* and Δ*BcHK67* mutants were approximately 10% shorter than those from the WT and ET strains, with no significant difference in width (Figure 4a,b). Conidial production was also markedly reduced in the mutants, decreasing by ~53–55% in the Δ*BcHK71* mutant and by ~28–29% in Δ*BcHK67* relative to WT and ET strains (Figure 4c). Additionally, mutant strains produced conidiophores with smaller clusters heads (Figure 4d,e). During infection-related development, conidia from the mutants exhibited delayed germination within the first 6 hpi (Figure 4f). Although AP formed by 10 hpi, their formation rates were lower in the mutants (Figure 4g). Germ tubes (GTs) produced by the mutants were significantly shorter at 9 hpi (Figure 4h,i). IC number and size on hydrophobic films were reduced in mutants at 24 and 48 hpi (Figure 5a–c). On onion epidermis, IC production was significantly lower in mutants at 48 hpi (Figure 5d,e). Collectively, these results indicate that *BcHK71* and *BcHK67* are required for normal asexual reproduction and the development of infection structures in *B. cinerea*, including conidiation, conidial germination, AP formation, and IC development.

### 3.5. BcHK71 and BcHK67 Are Virulence Determinants of B. cinerea

**Figure 6:**
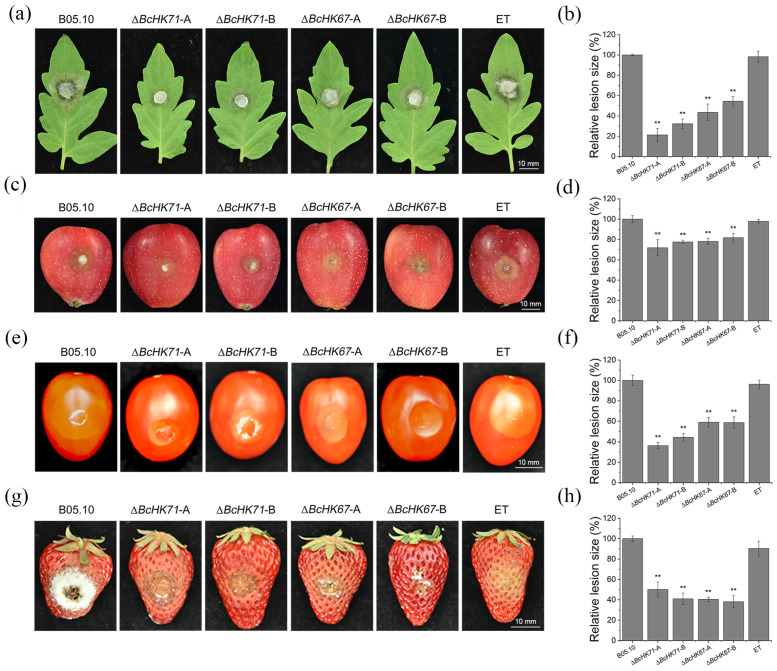
*BcHK71* and *BcHK67* are required for full virulence of *B. cinerea* on multiple host plants. (**a**,**c**,**e**,**g**) Representative disease symptoms caused by the WT, Δ*BcHK71*, Δ*BcHK67* mutants, and ET strains on wounded tomato leaves (**a**), apple fruits (**c**), tomato fruits (**e**), and strawberry fruits (**g**) at 3 dpi. (**b**,**d**,**f**,**h**) Quantification of lesion diameters corresponding to the panels on the left: tomato leaves (**b**), apple fruits (**d**), tomato fruits (**f**), and strawberry fruits (**h**). Data are presented as the mean ± standard deviation (SD) from three independent biological replicates (n = 3). ** *p* < 0.01 indicates a significant difference compared to the WT (Dunnett’s test).

Pathogenicity assays were conducted on tomato leaves as well as apple, tomato, and strawberry fruits. The deletion mutant (Δ*BcHK71*, Δ*BcHK67*) exhibited severe defects in infection process compared to the WT and ET strains. During early invasion, the mutants displayed delayed penetration and reduced colonization on all hosts (Figure 6a,c,e,g). Lesion sizes caused by the mutants were approximately 18% to 78% smaller than those produced by the WT and ET strains across all hosts (Figure 6b,d,f,h). These findings demonstrate that *BcHK71* and *BcHK67* are essential for full virulence in *B. cinerea*, playing critical roles in both host penetration and subsequent necrotrophic colonization.

### 3.6. Deletion of BcHK71 and BcHK67 Alters Stress Adaptation and Fungicide Sensitivity

**Figure 7:**
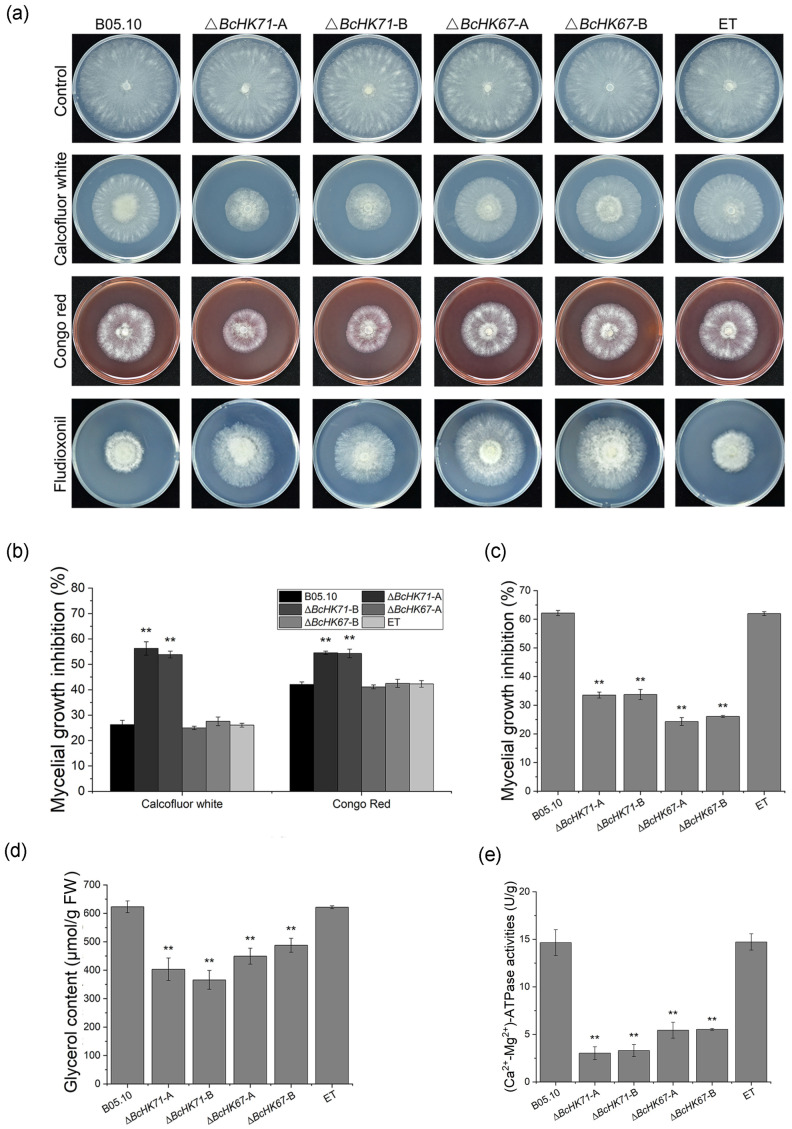
Deletion of *BcHK71* or *BcHK67* alters cell wall integrity, fungicide sensitivity, and glycerol metabolism. (**a**) Colony morphology of the WT, mutant (Δ*BcHK71*, Δ*BcHK67*), and ET strains on CM supplemented with 0.3 mg/mL Congo Red, 0.05 mg/mL Calcofluor White, or 0.00625 µg/mL fludioxonil. (**b**,**c**) Quantitative analysis of growth inhibition relative to the untreated control under cell wall stress (**b**) and fludioxonil treatment (**c**). (**d**) Intracellular glycerol content. (**e**) Ca^2+^-Mg^2+^-ATPase activity. All data are presented as the mean ± standard deviation (SD) from three independent biological replicates (n = 3). ** *p* < 0.01 indicates a significant difference compared to the WT strain (Dunnett’s test).

To evaluate the contribution of *BcHK71* and *BcHK67* to fungal stress adaptation, we assessed the tolerance of the mutants to various environmental stressors. Compared with the WT and ET strains, the Δ*BcHK71* mutant exhibited increased sensitivity to the cell wall-perturbing agents Congo Red and Calcofluor White, whereas the Δ*BcHK67* mutant showed no significant change in sensitivity (Figure 7a,b). Both mutants were less sensitive to fludioxonil and displayed reduced Ca^2+^-Mg^2+^-ATPase activity (Figure 7a,c,e). Glycerol content was also significantly lower in both mutants than WT and ET strains (Figure 7d). No significant differences in sensitivity to osmotic stressors (NaCl, KCl, sorbitol, glycerol), oxidative stressors (methyl viologen, H_2_O_2_), SDS, or CaCl_2_ were observed across WT, ET and mutant strains (Figure S3). These results suggest that *BcHK71* and *BcHK67* have distinct roles in maintaining fungal physiological balance in response to environmental stressors.

### 3.7. BcHK71 and BcHK67 Deletion Affects the Expression of Ypd1, Brrg1 and Skn7

**Figure 8:**
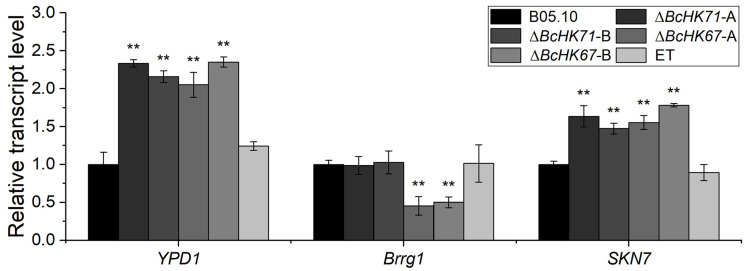
Relative expression of *BcYpd1*, *BcBrrg1* and *BcSkn7* in the WT, Δ*BcHK71*, Δ*BcHK67* and ET strains. Data are presented as mean ± SD (n = 3). Asterisks indicate significant differences compared to the WT: ** *p* < 0.01 vs. WT (Dunnett’s test).

To investigate the impact of *BcHK71* and *BcHK67* on downstream signaling components, we analyzed the transcript levels of genes (*BcYpd1*) and two RR genes *(BcBrrg1* and *BcSkn7*) using RT-qPCR. Compared with the WT and ET strains, *BcYpd1* was upregulated by approximately twofold in both Δ*BcHK71* and Δ*BcHK67* mutants. *BcBrrg1* expression remained unchanged in the Δ*BcHK71* mutant but was downregulated by approximately 50% in the Δ*BcHK67* mutant. In contrast, *BcSkn7* expression was upregulated by ~1.5-fold in both Δ*BcHK71* and Δ*BcHK67* mutants (Figure 8). These results suggest that *BcHK71* and *BcHK67* differentially regulate key components of the histidine kinase phosphorelay system.

### 3.8. Effect on BcHog1 Phosphorylation by BcHK71 and BcHK67

**Figure 9:**
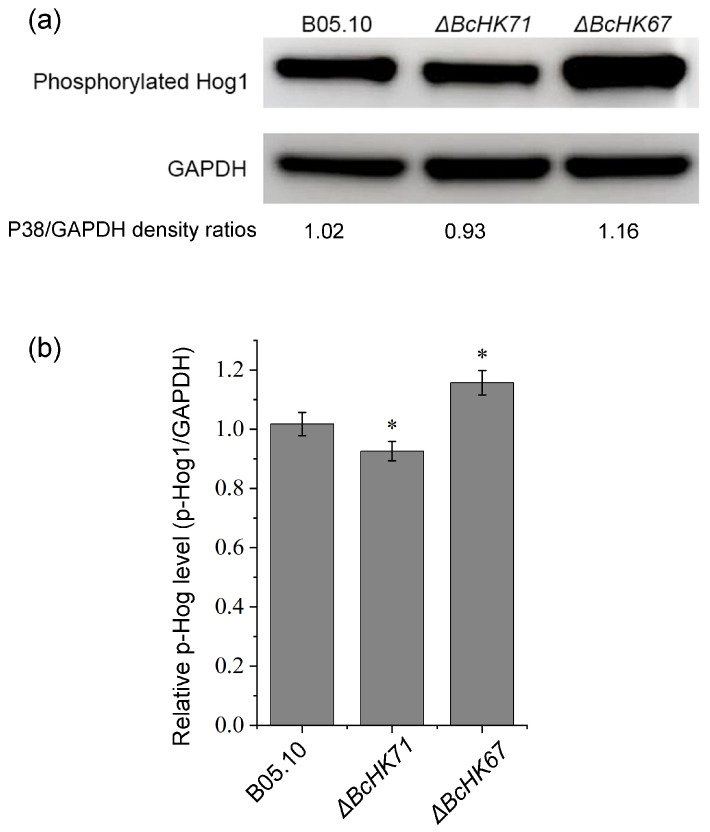
BcHK71 and BcHK67 differentially regulate BcHog1 phosphorylation. (**a**) Representative Western blot analysis of phosphorylated BcHog1 (p-BcHog1) and GAPDH (loading control) in the WT, Δ*BcHK71*, and Δ*BcHK67* strains after 2 day incubation in liquid complete medium. (**b**) Densitometric quantification of p-BcHog1 protein levels normalized to GAPDH. Data are presented as the mean ± SD (n = 3). * *p* < 0.05 indicates a significant difference versus the WT strain (Dunnett’s test).

To investigate the potential effects of the *BcHK71* and *BcHK67* deletion on phosphorylation of BcHog1, a key downstream component of the HOG-MAPK pathway, we assessed BcHog1 phosphorylation levels. This analysis was conducted using the representative Δ*BcHK71*-A and Δ*BcHK67*-A mutant strains. Densitometric analysis (normalized to GAPDH) showed a slight but statistically significant reduction in BcHog1 phosphorylation in Δ*BcHK71* (0.93 ± 0.03 vs. 1.02 ± 0.04 in WT, *p* < 0.05), and a markedly increased level in Δ*BcHK67* (1.16 ± 0.04, *p* < 0.05) (Figure 9). Together, these results suggest that BcHK67 negatively regulates of BcHog1 phosphorylation, whereas BcHK71 positive regulates it.

### 3.9. Transcriptomic Alterations in ΔBcHK71 and ΔBcHK67

To identify genes regulated by *BcHK71* and *BcHK67*, we performed RNA-seq analysis in WT, Δ*BcHK71*, and Δ*BcHK67* strains. Compared to the WT, the Δ*BcHK71* mutant exhibited 702 DEGs (|log_2_FC| ≥ 5, FDR < 0.001), including 356 upregulated and 346 downregulated genes (Figure S4a). In contrast, the Δ*BcHK67* mutant showed few transcriptional changes, with only 375 DEGs (|log_2_FC| ≥ 5, FDR < 0.001), comprising 195 upregulated and 180 downregulated genes (Figure S4b). GO enrichment analysis showed that the DEGs were significantly enriched in terms related to metabolic processes, cellular components, and catalytic activity (Figure S4c,d). In the Δ*BcHK71* mutant, KEGG pathway analysis identified the MAPK signaling pathway, ABC transporters, pentose and glucuronate interconversions, tryptophan metabolism, and tyrosine metabolism as the most significantly enriched pathways (Figure S5a). In the Δ*BcHK67* mutant, the top enriched pathways included the MAPK signaling pathway, carbon metabolism, starch and sucrose metabolism, and tryptophan metabolism (Figure S5b). Overall, these results indicate that both *BcHK71* and *BcHK67* have broad regulatory roles in diverse biochemical processes essential for normal growth, development, and virulence in *B. cinerea*.

### 3.10. Transcriptional Alterations in the Botrydial Biosynthetic Gene Cluster

We observed significant transcriptional changes in genes associated with phytotoxin biosynthesis. Notably, the entire botrydial biosynthetic gene cluster (*BcBOT1* to *BcBOT5*) was markedly upregulated in both Δ*BcHK71* and Δ*BcHK67* mutants compared with the WT strain (Figure S6). All five *BcBOT* genes showed consistently and significantly elevated expression levels (Table S3). To validate these results, we performed RT-qPCR analysis, which confirmed the strong upregulation of all *BcBOT* genes in both mutants (Table S3). Interestingly, this widespread introduction of phytotoxin biosynthetic genes occurred despite the pronounced reduction in virulence observed in the Δ*BcHK71* and Δ*BcHK67* mutants (as shown in Figure 6).

### 3.11. Expression of HOG-MAPK Pathway Genes in ΔBcHK71 and ΔBcHK67 Mutants

Given the close association of HOG-MAPK signaling with fungal response to environmental stresses, we analyzed DEGs within this pathway. As shown in Figure S6 and Table S3, the expression of most HOG-MAPK pathway genes was unaffected in both Δ*BcHK71* and Δ*BcHK67* mutants (Section 3.8, Figure 9). *BcBos4*, encoding a MAPKKK component, was the only gene upregulated in the Δ*BcHK71* mutant compared to the WT and Δ*BcHK67* strains. Other core components, including *BcBos5* (MAPKK), *BcSak1* (MAPK), and *BcPtpA* (a phosphatase that dephosphorylates Hog1), exhibited consistent expression levels across all strains (Figure S6 and Table S3). These results indicate that deletion of *BcHK71* or *BcHK67* does not globally alter the transcription of the HOG-MAPK pathway. The observed modulation of BcHog1 phosphorylation by these HKs (Figure 9) is therefore likely mediated through post-translational mechanisms rather than transcriptional regulation of the core pathway components.

## 4. Discussion

The fungal two-component signaling system is an evolutionarily conserved regulatory framework that governs key physiological processes, including morphogenesis, developmental programming, and environmental adaptation [57]. HKs act as upstream sensors that modulate fungal growth, differentiation, and stress responses [58]. Notably, *S. cerevisiae* possesses only a single HKs, whereas *B. cinerea* encodes more than 20 HKs genes [13]. This diversification likely reflects an evolutionary adaptation to diverse ecological niches and host ranges, enhancing the pathogen’s ability to sense and respond to host-derived signals during infection.

### 4.1. Structural Domains of BcHK71 and BcHK67

Fungal HKs typically feature an N-terminal sensor domain, a central transmitter region with conserved HisKA and HATPase_c domains, and a C-terminal receiver domain (RD) [13,59]. Both BcHK71 and BcHK67 were predicted to contain nine transmembrane helices in their N-terminus, followed by PAS, HisKA, HATPase_c, and RR domains, consistent with previous reports [60]. Their classification into Group XI—often associated with sensing light and oxygen—together with the presence of PAS domains, suggests a potential role in perceiving environmental cues [61,62]. The PAS domain is a conserved sensing and signaling module across kingdoms [59,63,64]. In fungi, PAS domains of Group XI HKs resemble those in Group VIII but differ from the PAS variants found in Groups III and VI.

### 4.2. Phylogenetic Relationships and Evolutionary Implications

The phylogenetic tree of BcHK71 and BcHK67 showed that they clustered closely with their orthologs within Sclerotiniaceae, separate from those in Sordariomycetes (Figure 1). This observation is consistent with the expected divergence of orthologous genes through speciation. The distinct clustering within Sclerotiniaceae may suggest a conserved evolutionary trajectory for these HKs, potentially reflecting shared functional constraints or adaptive processes specific to this fungal family. However, we acknowledge that the evolutionary history of pathogenic fungi is complex, and this single-gene phylogeny cannot resolve mechanisms like host jumps or horizontal gene transfer. Therefore, while this phylogenetic pattern primarily describes the evolutionary relationships, the hypotheses regarding its functional and adaptive significance require further experimental validation.

### 4.3. Roles of BcHK71 and BcHK67 in Development

Disruption of *BcHK71* and *BcHK67* revealed their critical roles in fungal development. Mutants produced fewer and smaller sclerotia despite exhibiting mycelial growth and dry weight comparable to WT and ET strains, suggesting that these HKs regulate signaling pathways critical for sclerotogenesis in *B. cinerea*. These phenotypic defects were consistently observed in two independent knockout mutants for each gene, confirming that the impairments were direct consequences of gene deletion rather than off-target effects. Sclerotia serve as durable resting structures and female reproductive elements, whose fertility depends on fertilization by microconidia to form apothecia. Impaired sclerotial development in the mutants therefore indicates disrupted sexual differentiation, consistent with observations in the Δ*Bcskn7* mutant of *B. cinerea* strain 38B1 [24].

Deletion of *BcHK71* and *BcHK67* also significantly reduced conidial production due to impaired conidiophore cluster formation, highlighting a regulatory role for Group XI HKs in conidiation. Previous studies have shown that the HOG pathway sensors *Bcsho1* and *Bcsln1* regulate morphology in *B. cinerea* [65]. Evidence from cyanobacteria, where deletion of the histidine kinase gene *Cdgk* altered cell morphology [66], further supports the role of HKs as regulators of cellular structure. Thus, *BcHK71* and *BcHK67* likely contribute to maintaining conidial morphology in *B. cinerea*.

In addition, developmental processes critical for pathogenesis—including spore germination and AP-mediated host penetration and IC formation—were significantly delayed in Δ*BcHK71* and Δ*BcHK67* mutants. The ICs were fewer and smaller following gene deletion (Figure 5c,d). Similar virulence defects have been reported in *B. cinerea* lacking *BcSKN7*, *BcCRZ1*, *BcSAK1*, and *BcBMP1* [24,67,68,69].

### 4.4. BcHK71 and BcHK67 Are Required for Full Virulence

Pathogenicity assays on detached wound-inoculated tomato leaves, apple fruits, tomato fruits, and strawberry fruits showed that knockout of *BcHK71* and *BcHK67* resulted in significantly reduced pathogenicity in the mutants compared to the WT strain (Figure 6). These results highlight the critical role of Group XI HKs in *B. cinerea* pathogenicity. Microscopic observations and statistical analysis demonstrated that GT formation, AP development and IC differentiation were delayed in the Δ*BcHK71* and Δ*BcHK67* mutants, highlighting the importance of these genes in morphogenesis (Figure 4f–i and Figure 5b–e). Although our pathogenicity assays involved wound inoculation, the Δ*BcHK71* and Δ*BcHK67* mutants still exhibited reduced virulence compared to the WT and ET strains, suggesting that the impaired lesion expansion is not solely a consequence of defective penetration. Instead, these findings suggest that *BcHK71* and *BcHK67* are critical for post-invasive host colonization. The results are consistent with *bos1*-null mutants in *B. cinerea*, which also showed reduced pathogenicity [70]. In contrast, knockout of *HIK1* in *M. oryzae* did not affect pathogenicity [23]. These findings indicate functional divergence among HKs in regulating virulence strategies across fungal pathogens, supporting their potential as targets for antifungal strategies.

*B. cinerea* produces phytotoxic compounds such as botrydial (a sesquiterpene) and botcinic acid (a polyketide), which contribute to virulence. Notably, both Δ*BcHK71* and Δ*BcHK67* mutants exhibited coordinated upregulation of the entire botrydial biosynthetic gene cluster (*BcBOT1*–*BcBOT5*) despite reduced virulence [71,72]. This result was consistently confirmed by RNA-seq and qPCR analyses (Figure S6, Table S3). This disconnect challenges the assumption that increased pathogenicity gene expression always enhances virulence [73]. The precise molecular mechanisms underlying the attenuated virulence in Δ*BcHK71* and Δ*BcHK67* mutants warrant further investigation. Our transcriptome data, which revealed broad transcriptional dysregulation in the mutants (Figures S4 and S5), suggest that a global disruption of the regulatory network may contribute to the observed phenotypic defects. Future work, such as direct measurement of enzyme activities and targeted metabolomic profiling of botrydial, will be essential to dissect the exact contribution of these pathways to the virulence orchestrated by *BcHK71* and *BcHK67*.

### 4.5. Roles of BcHK71 and BcHK67 Under Various Stresses

The penetration defects observed in the mutant strains may result from impaired cell wall remodeling, compromising cell wall integrity. The fungal cell wall, composed of chitin, glucan, and mannoproteins, is essential for maintaining cell shape, nutrient exchange and virulence [74,75,76]. Remodeling during growth and infection is critical for pathogenicity. In *Candida albicans*, *CHK1*-null mutants showed increased sensitivity to Congo Red and altered cell wall composition [77], while *Aspergillus nidulans NikA* and *BcSkn7* mutants exhibited sensitivity to Calcofluor White [23,78]. In this study, the Δ*BcHK71* mutant displayed increased sensitivity to Congo Red, Calcofluor White, whereas the Δ*BcHK67* mutant did not, underscoring their distinct roles in maintaining cell wall integrity. Transcriptome analysis further revealed greater disruption of genes related to cell wall/membrane/envelope biogenesis in Δ*BcHK71* than in Δ*BcHK67* (Figure S7a,b), aligning with the observed cell wall integrity defects (Figure 7a,b). KEGG analysis also identified six differentially expressed genes in the MAPK signaling pathway in the Δ*BcHK71* (Figure S5a).

In *N. crassa*, *Os-1* deletion mutant exhibited increased sensitivity to high salt and sugar stress, while the *Os-2* mutant was sensitive to NaCl [79,80]. In *B. cinerea*, deletion of *BcSkn7* increased sensitivity to hydrogen peroxide, contrasting with the responses of Δ*BcHK67* and Δ*BcHK71* mutants (Figure S3) [24]. These findings indicate that other HKs, rather than *BcHK71* or *BcHK67*, mediate osmotic and oxidative stress responses in *B. cinerea*, consistent with specialized roles of two-component signaling in oxidative stress adaptation [81,82,83].

### 4.6. BcHK71 and BcHK67 Influence Fungicide Sensitivity and Glycerol Biosynthesis

Fludioxonil, a phenylpyrrole fungicide widely used in post-harvest protection [84], targets Group III hybrid HKs (HHK3, e.g., Os-1) [85]. Binding of fludioxonil aberrantly activates the HOG-MAPK pathway, resulting in Hog1 phosphorylation (p-Hog1), excessive glycerol synthesis, and growth inhibition [86,87]. Field resistance to fludioxonil is rare and is primarily associated with HHK3 mutations in fungi such as *N. crassa* and *B. cinerea* [88]. Key HOG pathway components include osmosensor HKs (e.g., OS-1) for osmotic sensing [89,90,91] and the terminal MAPK Hog1, which drives glycerol biosynthesis upon phosphorylation [92].

Mutations in Group III HKs are the predominant mechanism of fludioxonil resistance, although mutations in other genes or overexpression of efflux transporters may also contribute [15,88]. In *N. crassa*, resistance-conferring mutations occur in *Os-2*, which encodes a MAP kinase in the HOG pathway. Deletion mutants of *Os-2*, *Os-4*, and *Os-5* confers resistance to both iprodione and fludioxonil [93]. Similarly, disruption of *FgOS-1*, *FgOS-2*, *FgOS-4,* or *FgOS-5* in *F. graminearum* confers resistance to both fungicides [94]. In *B. cinerea*, knockout of *BcSKN7*, *BcBOS1* and *BcBrrg-1* reduced fludioxonil susceptibility [25,95,96].

In *B. cinerea*, Δ*BcHK6*7 and Δ*BcHK7*1 mutants did not exhibit increased fludioxonil sensitivity or a hyperosmotic stress response but showed reduced glycerol accumulation. These findings suggest a distinct role for the two-component system in phenylpyrrole sensitivity in *B. cinerea* compared with other filamentous fungi. Given that HKs are generally essential for stress responses, their deletion often incurs fitness costs, including growth defects and osmosensitivity [97,98]. The absence of such typical phenotypes in our mutants further underscores the uniqueness of BcHK67 and BcHK71 function. Beyond the direct roles of HKs, alternative mechanisms can confer fungicide resistance. For instance, efflux-mediated multidrug resistance (MDR) in *B. cinerea* enables active fungicide efflux and is not specific to fludioxonil. This mechanism can be achieved through distinct pathways, such as the overexpression of the ABC transporter BcAtrB [99,100] or upregulation of the MFS transporter BcMfsM2 [101]. Consistently, Δ*BcatrB* mutants demonstrate heightened sensitivity to fludioxonil, whereas strains overexpressing *BcatrB* exhibit reduced sensitivity [99]. Future studies should investigate whether overexpression of BcHK71 or BcHK67 confers fludioxonil resistance.

### 4.7. BcHK71 and BcHK67 Are Required for Downstream Signaling in the Two-Component System

The HOG pathway in *S. cerevisiae* is among the best-characterized eukaryotic two-component systems. Its genome encodes the sensor HKs Sln1, the HPt protein Ypd1, and two RRs, Ssk1 and Skn7 [19,20]. Under normal osmotic conditions, active Sln1 autophosphorylates and transfers a phosphate to Ypd1, which then phosphorylates Ssk1 [102]. Similarly, *B. cinerea* —like yeast and other filamentous fungi —possesses two orthologous RRs, BcBrrg1 (a functional homolog of Ssk1) and BcSkn7, as well as a single Hpt protein, BcYpd1 [103].

Our findings indicate that the knockout of *BcHK71* and *BcHK67* upregulates *BcYpd1* expression, suggesting that compensatory regulation by the other HKs. *BcHK71* deletion did not affect *BcBrrg1* expression but increased *BcSkn7* expression, implying that signaling primarily proceeds through *BcSkn7*. In contrast, *BcHK67* knockout decreased *BcBrrg1* expression while increasing *BcSkn7* expression, highlighting a central key role for *BcSkn7* in signaling after *BcHK67* loss.

Under hyperosmotic conditions, inhibition of Sln1 activity leads to the accumulation of unphosphorylated Ssk1, which activates the downstream MAP kinase cascade (Ssk2/Ssk22-Pbs2-Hog1). Phosphorylated Hog1 translocates to the nucleus, where it regulates transcription factors that induce glycerol synthesis to maintain cell turgor. Upon restoration of homeostasis, protein phosphatases dephosphorylate Hog1, re-establishing osmotic equilibrium [104].

Homologs of the yeast HOG-MAPK pathway in *B. cinerea* regulate growth, development, differentiation, and responses to oxidative, osmotic, fungicidal, and cell wall stresses [96]. BcSak1, the orthologous of Hog1, becomes phosphorylated under oxidative and osmotic stress, fungicide exposure, and Calcofluor White treatment [68]. In this study, Δ*BcHK71* exhibited increased sensitivity to Calcofluor White and Congo Red, whereas Δ*BcHK67* did not. The distinct phenotypes of the mutants may be linked to differential phosphorylation status of BcHog1: *BcHK67* deletion significantly increased Hog1 phosphorylation, while *BcHK71* deletion caused only a slight reduction (Figure 9). This suggests that *BcHK67* acts as a strong negative regulator of the HOG pathway, whereas *BcHK71* plays a minor positive regulatory role.

Transcriptomic analysis showed that most HOG–MAPK pathway genes, including *BcHog1*, were not differentially expressed in either mutant (Section 3.11, Table S3). This indicates that *BcHK71* and *BcHK67* modulate BcHog1 phosphorylation at the post-translational level rather than through transcriptional regulation of core components. The upregulation of *BOS4* (MAPKKK) specifically in the Δ*BcHK71* mutant may represent a compensatory feedback mechanism, although it was insufficient to restore Hog1 phosphorylation to WT levels. Thus, BcHK71 and BcHK67 likely fine-tune the HOG through post-translational interactions with upstream sensors or downstream components. The opposing effects on Hog1 phosphorylation warrant further investigation, and other HKs may compensate for their loss in *B. cinerea*.

### 4.8. Distinctive Role of Group XI HKs in Virulence Regulation

Our findings position Group XI HKs as specialized virulence regulators, distinct from other HK groups. First, the profound impairment in sclerotia production, germination, appressoria, and cell wall integrity in Δ*BcHK71* and Δ*BcHK67* mutants underscores a role that extends beyond the general stress adaptation functions of Group III HKs. Second, we identify a novel, fungus-specific mechanism: *BcHK67* serves as a critical upstream node that fine-tunes the HOG-MAPK pathway and botrydial production, a regulatory link not observed in Group XI orthologs of other species. This work is the first to decrypt how Group XI HKs in *B. cinerea* orchestrate a complex pathogenicity network, offering a new paradigm for understanding fungal two-component systems.

### 4.9. Conclusions

**Figure 10:**
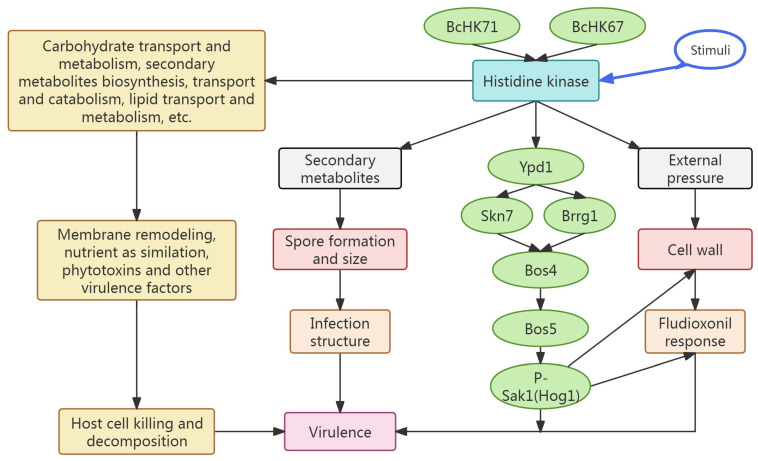
A proposed model summarizing the roles of *BcHK71* and *BcHK67* in *B. cinerea*. This schematic integrates the key findings of this study, illustrating how these histidine kinases regulate the HOG-MAPK pathway, vegetative growth, development, and virulence. Solid lines represent regulatory relationships supported by the data in this study, while dashed lines indicate proposed or previously established interactions.

This study establishes that *B. cinerea* HKs *BcHK71* and *BcHK67* are essential for pathogenicity, regulating virulence gene expression, vegetative growth, and development. *BcHK71* also maintains cell wall integrity. Both genes influence fludioxonil sensitivity, suggesting roles in fungicide resistance. The functions of these HKs may have broader implications for fungal virulence, a premise that warrants validation in other phytopathogens (Figure 10). Future studies should evaluate their roles in field-evolved resistance, elucidating the underlying mechanisms in natural populations, and explore their potential as targets for novel antifungal strategies.

## Data Availability

The data presented in this study are available on request from the corresponding author. These data will not be made public due to privacy or ethical constraints.

## Supplementary Materials

The following supporting information can be downloaded at: https://www.mdpi.com/article/10.3390/jof11120850/s1, Figure S1: Multiple sequence alignment of BcHK71 and its homologs. Figure S2: Multiple sequence alignment of BcHK67 and its homologs. Figure S3: Response of *B. cinerea* histidine kinase mutants to abiotic stress. Figure S4: Transcriptomic profiles of Δ*BcHK71* and Δ*BcHK67* mutants. Figure S5: KEGG pathway enrichment analysis of DEGs. Figure S6: Expression heatmap of selected differentially expressed genes. DEGs Figure S7: Functional categorization of DEGs using the COG database. Table S1: Primers used in this study. Table S2: Protein sequences used in the phylogenetic analysis of BcHK71 and BcHK67. Table S3: Comparison in the changes of gene expression determined by DESeq2 sequencing and qPCR approaches. Supplementary Dataset S1: Complete dataset of the Gene Ontology (GO) enrichment analysis.

## References

[1] Fillinger S., Elad Y. (2016). Botrytis-the Fungus, the Pathogen and Its Management in Agricultural Systems.

[2] Weiberg A., Wang M., Lin F.M., Zhao H., Zhang Z., Kaloshian I., Huang H.-D., Jin H. (2013). Fungal small RNAs suppress plant immunity by hijacking host RNA interference pathways. Science.

[3] Dean R., Van Kan J.A., Pretorius Z.A., Hammond-Kosack K.E., Di Pietro A., Spanu P.D., Dickman M., Kahmann R., Ellis J., Foster G.D. (2012). The Top 10 fungal pathogens in molecular plant pathology. Mol. Plant Pathol..

[4] Liu J.K., Chang H.W., Liu Y., Qin Y., Ding Y.H., Wang L., Zhao Y., Zhang M.-Z., Cao S.-N., Li L.-T. (2018). The key gluconeogenic gene PCK1 is crucial for virulence of *Botrytis cinerea* via initiating its conidial germination and host penetration. Environ. Microbiol..

[5] Choquer M., Rascle C., Gonçalves I.R., de Vallée A., Ribot C., Loisel E., Smilevski P., Ferria J., Savadogo M., Souibgui E. (2021). The infection cushion of *Botrytis cinerea*: A fungal ‘weapon’ of plant-biomass destruction. Environ. Microbiol..

[6] Van Kan J.A., Marissen N., van Doorn W.G., van Meeteren U. (2003). Infection strategies of *Botrytis cinerea*. VIII International Symposium on Postharvest Physiology of Ornamental Plants 669.

[7] Capra E.J., Laub M.T. (2012). Evolution of two-component signal transduction systems. Annu. Rev. Microbiol..

[8] Ji S., Li C., Liu M., Liu Y., Jiang L. (2024). Targeting new functions and applications of bacterial two-component systems. ChemBioChem.

[9] Koretke K.K., Lupas A.N., Warren P.V., Rosenberg M., Brown J.R. (2000). Evolution of two-component signal transduction. Mol. Biol. Evol..

[10] Appleby J.L., Parkinson J.S., Bourret R.B. (1996). Signal transduction via the multi-step phosphorelay: Not necessarily a road less traveled. Cell.

[11] Quinn J., Malakasi P., Smith D.A., Cheetham J., Buck V., Millar J.B., Morgan B.A. (2011). Two-component mediated peroxide sensing and signal transduction in fission yeast. Antioxid. Redox Signal..

[12] Yoshimi A., Hagiwara D., Ono M., Fukuma Y., Midorikawa Y., Furukawa K., Fujioka T., Mizutani O., Sato N., Miyazawa K. (2021). Downregulation of the *ypdA* gene encoding an intermediate of His-Asp phosphorelay signaling in *Aspergillus nidulans* induces the same cellular effects as the phenylpyrrole fungicide fludioxonil. Front. Fungal Biol..

[13] Catlett N.L., Yoder O.C., Turgeon B.G. (2003). Whole-genome analysis of two-component signal transduction genes in fungal pathogens. Eukaryot. Cell.

[14] Kushwaha H.R., Singla-Pareek S.L., Pareek A. (2014). Putative osmosensor-OsHK3b-a histidine kinase protein from rice shows high structural conservation with its ortholog AtHK1 from *Arabidopsis*. J. Biomol. Struct. Dyn..

[15] Bourret R.B., Borkovich K.A., Simon M.I. (1991). Signal transduction pathways involving protein phosphorylation in prokaryotes. Annu. Rev. Biochem.

[16] Alvarez A.F., Georgellis D. (2022). The role of sensory kinase proteins in two-component signal transduction. Biochem. Soc. Trans..

[17] Liao B., Ye X., Chen X., Zhou Y., Cheng L., Zhou X., Ren B. (2021). The two-component signal transduction system and its regulation in *Candida albicans*. Virulence.

[18] West A.H., Stock A.M. (2001). Histidine kinases and response regulator proteins in two-component signaling systems. Trends Biochem. Sci..

[19] Posas F., Wurgler-Murphy S.M., Maeda T., Witten E.A., Thai T.C., Saito H. (1996). Yeast HOG1 MAP kinase cascade is regulated by a multistep phosphorelay mechanism in the SLN1–YPD1–SSK1 “two-component” osmosensor. Cell.

[20] Kaserer A.O., Andi B., Cook P.F., West A.H. (2009). Effects of osmolytes on the SLN1-YPD1-SSK1 phosphorelay system from *Saccharomyces cerevisiae*. Biochemistry.

[21] Saito H. (2001). Histidine phosphorylation and two-component signaling in eukaryotic cells. Chem. Rev..

[22] Rispail N., Soanes D.M., Ant C., Czajkowski R., Grünler A., Huguet R., Perez-Nadales E., Poli A., Sartorel E., Valiante V. (2009). Comparative genomics of MAP kinase and calcium-calcineurin signalling components in plant and human pathogenic fungi. Fungal Genet. Biol..

[23] Motoyama T., Kadokura K., Ohira T., Ichiishi A., Fujimura M., Yamaguchi I., Kudo T. (2005). A two-component histidine kinase of the rice blast fungus is involved in osmotic stress response and fungicide action. Fungal Genet. Biol..

[24] Viefhues A., Schlathoelter I., Simon A., Viaud M., Tudzynski P. (2015). Unraveling the function of the response regulator BcSkn7 in the stress signaling network of *Botrytis cinerea*. Eukaryot. Cell.

[25] Yoshimi A., Kojima K., Takano Y., Tanaka C. (2005). Group III histidine kinase is a positive regulator of Hog1-type mitogen-activated protein kinase in filamentous fungi. Eukaryot. Cell.

[26] Dexter J.P., Xu P., Gunawardena J., McClean M.N. (2015). Robust network structure of the Sln1-Ypd1-Ssk1 three-component phospho-relay prevents unintended activation of the HOG MAPK pathway in *Saccharomyces cerevisiae*. BMC Syst. Biol..

[27] Gu Q., Chen Y., Liu Y., Zhang C., Ma Z. (2015). The transmembrane protein FgSho1 regulates fungal development and pathogenicity via the MAPK module Ste50-Ste11-Ste7 in *Fusarium graminearum*. New Phytol..

[28] Xu M., Wang Q., Wang G., Zhang X., Liu H., Jiang C. (2022). Combatting Fusarium head blight: Advances in molecular interactions between *Fusarium graminearum* and wheat. Phytopathol. Res..

[29] Yang Y., Huang P., Ma Y., Jiang R., Jiang C., Wang G. (2022). Insights into intracellular signaling network in *Fusarium* species. Int. J. Biol. Macromol..

[30] Viaud M., Fillinger S., Liu W., Polepalli J.S., Le Pêcheur P., Kunduru A.R., Leroux P., Legendre L. (2006). A class III histidine kinase acts as a novel virulence factor in *Botrytis cinerea*. Mol. Plant-Microbe Interact..

[31] Ren W., Liu N., Sang C., Shi D., Zhou M., Chen C., Qin Q., Chen W. (2018). The autophagy gene *BcATG8* regulates the vegetative differentiation and pathogenicity of *Botrytis cinerea*. Appl. Environ. Microbiol..

[32] Rho H.S., Kang S., Lee Y.H. (2001). *Agrobacterium tumefaciens*-mediated transformation of the plant pathogenic fungus, *Magnaporthe grisea*. Mol. Cells.

[33] Tamura K., Stecher G., Kumar S. (2021). MEGA11: Molecular evolutionary genetics analysis version 11. Mol. Biol. Evol..

[34] Nichloas N.B. (1997). GeneDoc: Analysis and visualization of genetic variation. EMBnet News.

[35] Saitou N., Nei M. (1987). The neighbor-joining method: A new method for reconstructing phylogenetic trees. Mol. Biol. Evol..

[36] El-Gebali S., Mistry J., Bateman A., Eddy S.R., Luciani A., Potter S.C., Qureshi M., Richardson L.J., Salazar G.A., Smart A. (2019). The Pfam protein families database in 2019. Nucleic Acids Res..

[37] Schultz J., Copley R.R., Doerks T., Ponting C.P., Bork P. (2000). SMART: A web-based tool for the study of genetically mobile domains. Nucleic Acids Res..

[38] Ren J., Wen L., Gao X., Jin C., Xue Y., Yao X. (2009). DOG 1.0: Illustrator of protein domain structures. Cell Res..

[39] Li L., Wang J., Zhang Z., Wang Y., Liu M., Jiang H., Chai R., Mao X., Qiu H., Liu F. (2014). *MoPex19*, which is essential for maintenance of peroxisomal structure and woronin bodies, is required for metabolism and development in the rice blast fungus. PLoS ONE.

[40] Livak K.J., Schmittgen T.D. (2001). Analysis of relative gene expression data using real-time quantitative PCR and the 2^−ΔΔCT^ method. Methods.

[41] Li L., Yu M.X., Guo J., Hao Z.N., Zhang Z., Lu Z.Q., Wang J.Y., Zhu X.M., Wang Y.L., Chen J. (2022). The peroxins BcPex8, BcPex10, and BcPex12 are required for the development and pathogenicity of *Botrytis cinerea*. Front. Microbiol..

[42] Sinclair J.B., Dhingra O.D. (2017). Basic Plant Pathology Methods.

[43] Collins T.J. (2007). ImageJ for microscopy. Biotechniques.

[44] Sherman F. (2002). Getting started with yeast. Methods Enzymol..

[45] Martin M. (2011). Cutadapt removes adapter sequences from high-throughput sequencing reads. EMBnet. J..

[46] Pertea M., Kim D., Pertea G.M., Leek J.T., Salzberg S.L. (2016). Transcript-level expression analysis of RNA-seq experiments with HISAT, StringTie and Ballgown. Nat. Protoc..

[47] Pertea M., Pertea G.M., Antonescu C.M., Chang T.C., Mendell J.T., Salzberg S.L. (2015). StringTie enables improved reconstruction of a transcriptome from RNA-seq reads. Nat. Biotechnol..

[48] Love M.I., Huber W., Anders S. (2014). Moderated estimation of fold change and dispersion for RNA-seq data with DESeq2. Genome Biol..

[49] Kanehisa M., Goto S. (2000). KEGG: Kyoto encyclopedia of genes and genomes. Nucleic Acids Res..

[50] Gene Ontology Consortium (2019). The gene ontology resource: 20 years and still GOing strong. Nucleic Acids Res..

[51] Kolde R., Kolde M.R. (2015). Package ‘pheatmap’. R Package.

[52] Wickham H., Chang W., Wickham M.H. (2016). Package ‘ggplot2’. Create elegant data visualisations using the grammar of graphics. Version.

[53] Rozen S., Skaletsky H., Krawetz S., Misener S. (2000). Primer3 on the WWW for general users and for biologist programmers. Bioinformatics Methods and Protocols.

[54] Bergeron B.P., Rouse R.G. (1994). Imageview: A High-Level Authoring Tool for Repurposing Multimedia Content. J. Educ. Comput. Res..

[55] Dunnett C.W. (1955). A multiple comparison procedure for comparing several treatments with a control. J. Am. Stat. Assoc..

[56] Tang Q.Y., Zhang C.X. (2013). Data Processing System (DPS) software with experimental design, statistical analysis and data mining developed for use in entomological research. Insect Sci..

[57] Papon N., Stock A.M. (2019). Two-component systems. Curr. Biol..

[58] Fassler J.S., West A.H. (2013). Histidine phosphotransfer proteins in fungal two-component signal transduction pathways. Eukaryot. Cell.

[59] Hérivaux A., So Y.S., Gastebois A., Latgé J.P., Bouchara J.P., Bahn Y.S., Papon N. (2016). Major sensing proteins in pathogenic fungi: The hybrid histidine kinase family. PLoS Pathog..

[60] Defosse T.A., Sharma A., Mondal A.K., Dugé de Bernonville T., Latgé J.P., Calderone R., Giglioli-Guivarc N., Courdavault V., Clastre M., Papon N. (2015). Hybrid histidine kinases in pathogenic fungi. Mol. Microbiol..

[61] Paredes-Martínez F., Eixerés L., Zamora-Caballero S., Casino P. (2024). Structural and functional insights underlying recognition of histidine phosphotransfer protein in fungal phosphorelay systems. Commun. Biol..

[62] Shin J.H., Gumilang A., Kim M.J., Han J.H., Kim K.S. (2019). A PAS-containing histidine kinase is required for conidiation, appressorium formation, and disease development in the rice blast fungus, *Magnaporthe oryzae*. Mycobiology.

[63] Stuffle E.C., Johnson M.S., Watts K.J. (2021). PAS domains in bacterial signal transduction. Curr. Opin. Microbiol..

[64] Taylor B.L., Zhulin I.B. (1999). PAS domains: Internal sensors of oxygen, redox potential, and light. Microbiol. Mol. Biol. Rev..

[65] Ren W., Liu N., Yang Y., Yang Q., Chen C., Gao Q. (2019). The sensor proteins BcSho1 and BcSln1 are involved in, though not essential to, vegetative differentiation, pathogenicity and osmotic stress tolerance in *Botrytis cinerea*. Front. Microbiol..

[66] Sun Q.X., Huang M., Zhang J.Y., Zeng X., Zhang C.C. (2023). Control of cell size by c-di-GMP requires a two-component signaling system in the cyanobacterium *Anabaena* sp. strain PCC 7120. Microbiol. Spectr..

[67] Schumacher J., de Larrinoa I.F., Tudzynski B. (2008). Calcineurin-responsive zinc finger transcription factor CRZ1 of *Botrytis cinerea* is required for growth, development, and full virulence on bean plants. Eukaryot. Cell.

[68] Segmüller N., Ellendorf U., Tudzynski B., Tudzynski P. (2007). BcSAK1, a stress-activated mitogen-activated protein kinase, is involved in vegetative differentiation and pathogenicity in *Botrytis cinerea*. Eukaryot. Cell.

[69] Zheng L., Campbell M., Murphy J., Lam S., Xu J.R. (2000). The BMP1 gene is essential for pathogenicity in the gray mold fungus *Botrytis cinerea*. Mol. Plant-Microbe Interact..

[70] Cui F., Li X., Wu W., Luo W., Wu Y., Brosché M., Overmyer K. (2022). Ectopic expression of *BOTRYTIS SUSCEPTIBLE1* reveals its function as a positive regulator of wound-induced cell death and plant susceptibility to *Botrytis*. Plant Cell..

[71] Simon A., Dalmais B., Morgant G., Viaud M. (2013). Screening of a *Botrytis cinerea* one-hybrid library reveals a Cys2His2 transcription factor involved in the regulation of secondary metabolism gene clusters. Fungal Genet. Biol..

[72] Dalmais B., Schumacher J., Moraga J., Le Pecheur P., Tudzynski B., Collado I.G., Viaud M. (2011). The *Botrytis cinerea* phytotoxin botcinic acid requires two polyketide synthases for production and has a redundant role in virulence with botrydial. Mol. Plant Pathol..

[73] Van Kan J.A., Stassen J.H., Mosbach A., Van Der Lee T.A., Faino L., Farmer A.D., Papasotiriou D.G., Zhou S., Seidl M.F., Cottam E. (2017). A gapless genome sequence of the fungus *Botrytis cinerea*. Mol. Plant Pathol..

[74] Free S.J. (2013). Fungal cell wall organization and biosynthesis. Adv. Genet..

[75] Iqbal K., Yahya S., Jadoon M., Yaseen E., Nadeem Z. (2024). Strategies for cadmium remediation in nature and their manipulation by molecular techniques: A comprehensive review. Int. J. Environ. Sci. Technol..

[76] Lipke P.N., Ovalle R. (1998). Cell wall architecture in yeast: New structure and new challenges. J. Bacteriol..

[77] Navarro-Arias M.J., Dementhon K., Defosse T.A., Foureau E., Courdavault V., Clastre M., Le Gal S., Nevez G., Le Govic Y., Bouchara J.-P. (2017). Group X hybrid histidine kinase Chk1 is dispensable for stress adaptation, host-pathogen interactions and virulence in the opportunistic yeast *Candida guilliermondii*. Res. Microbiol..

[78] Vargas-Pérez I., Sánchez O., Kawasaki L., Georgellis D., Aguirre J. (2007). Response regulators SrrA and SskA are central components of a phosphorelay system involved in stress signal transduction and asexual sporulation in *Aspergillus nidulans*. Eukaryot. Cell.

[79] Makoto F., Noriyuki O., Akihiko I., Ron U., Koki H., Isamu Y. (2000). Sensitivity to phenylpyrrole fungicides and abnormal glycerol accumulation in os and cut mutant strains of *Neurospora crassa*. J. Pestic. Sci..

[80] Zhang Y., Lamm R., Pillonel C., Lam S., Xu J.R. (2002). Osmoregulation and fungicide resistance: The *Neurospora crassa* os-2 gene encodes a HOG1 mitogen-activated protein kinase homologue. Appl. Environ. Microbiol..

[81] Bahn Y.S. (2008). Master and commander in fungal pathogens: The two-component system and the HOG signaling pathway. Eukaryot. Cell.

[82] Tanaka C., Izumitsu K. (2010). Two-component signaling system in filamentous fungi and the mode of action of dicarboximide and phenylpyrrole fungicides. Fungicides.

[83] Corran A., Knauf-Beiter G., Zeun R. (2008). Fungicides acting on signal transduction. Modern Crop Protection Compounds.

[84] Yan L., Yang Q., Sundin G.W., Li H., Ma Z. (2010). The mitogen-activated protein kinase kinase BOS5 is involved in regulating vegetative differentiation and virulence in *Botrytis cinerea*. Fungal Genet. Biol..

[85] Diskin S., Sharir T., Feygenberg O., Maurer D., Alkan N. (2019). Fludioxonil-A potential alternative for postharvest disease control in mango fruit. Crop Prot..

[86] Jacoby T., Flanagan H., Faykin A., Seto A.G., Mattison C., Ota I. (1997). Two protein-tyrosine phosphatases inactivate the osmotic stress response pathway in yeast by targeting the mitogen-activated protein kinase, Hog1. J. Biol. Chem..

[87] Kilani J., Fillinger S. (2016). Phenylpyrroles: 30 years, two molecules and (nearly) no resistance. Front. Microbiol..

[88] Furukawa K., Randhawa A., Kaur H., Mondal A.K., Hohmann S. (2012). Fungal fludioxonil sensitivity is diminished by a constitutively active form of the group III histidine kinase. FEBS Lett..

[89] Schumacher M.M., Enderlin C.S., Selitrennikoff C.P. (1997). The osmotic-1 locus of *Neurospora crassa* encodes a putative histidine kinase similar to osmosensors of bacteria and yeast. Curr. Microbiol..

[90] Alex L.A., Borkovich K.A., Simon M.I. (1996). Hyphal development in *Neurospora crassa*: Involvement of a two-component histidine kinase. Proc. Natl. Acad. Sci. USA.

[91] Miller T.K., Renault S., Selitrennikoff C.P. (2002). Molecular dissection of alleles of the osmotic-1 locus of *Neurospora crassa*. Fungal Genet. Biol..

[92] Brewster J.L., de Valoir T., Dwyer N.D., Winter E., Gustin M.C. (1993). An osmosensing signal transduction pathway in yeast. Science.

[93] Oshima M., Banno S., Okada K., Takeuchi T., Kimura M., Ichiishi A., Yamaguchi I. (2006). Survey of mutations of a histidine kinase gene *BcOS1* in dicarboximide-resistant field isolates of *Botrytis cinerea*. J. Gen. Plant Pathol..

[94] Ochiai N., Tokai T., Nishiuchi T., Takahashi-Ando N., Fujimura M., Kimura M. (2007). Involvement of the osmosensor histidine kinase and osmotic stress-activated protein kinases in the regulation of secondary metabolism in *Fusarium graminearum*. Biochem. Biophys. Res. Commun..

[95] Yin X., Li P., Wang Z., Wang J., Fang A., Tian B., Yang Y., Yu Y., Bi C. (2024). Binding mode and molecular mechanism of the two-component histidine kinase Bos1 of *Botrytis cinerea* to Fludioxonil and Iprodione. Phytopathology.

[96] Yan L., Yang Q., Jiang J., Michailides T.J., Ma Z. (2011). Involvement of a putative response regulator *Brrg-1* in the regulation of sporulation, sensitivity to fungicides, and osmotic stress in *Botrytis cinerea*. Appl. Microbiol. Biotechnol..

[97] Hagiwara D., Matsubayashi Y., Marui J., Furukawa K., Yamashino T., Kanamaru K., Kato M., Abe K., Kobayashi T., Mizuno T. (2007). Characterization of the NikA histidine kinase implicated in the phosphorelay signal transduction of *Aspergillus nidulans*, with special reference to fungicide responses. Biosci. Biotechnol. Biochem..

[98] Sofianos G., Piombo E., Dubey M., Karlsson M., Karaoglanidis G., Tzelepis G. (2024). Transcriptomic and functional analyses on a *Botrytis cinerea* multidrug-resistant (MDR) strain provides new insights into the potential molecular mechanisms of MDR and fitness. Mol. Plant Pathol..

[99] SVermeulen T., Schoonbeek H., De Waard M.A. (2001). The ABC transporter BcatrB from *Botrytis cinerea* is a determinant of the activity of the phenylpyrrole fungicide fludioxonil. Pest Manag. Sci. Former. Pestic. Sci..

[100] Wu Z., Yu C., Bi Q., Zhang J., Hao J., Liu P., Liu X. (2024). Procymidone application contributes to multidrug resistance of *Botrytis cinerea*. J. Fungi.

[101] Liu M., Peng J., Wang X., Zhang W., Zhou Y., Wang H., Li X., Yan J., Duan L. (2023). Transcriptomic analysis of resistant and wild-type *Botrytis cinerea* isolates revealed fludioxonil-resistance mechanisms. Int. J. Mol. Sci..

[102] Hohmann S. (2002). Osmotic stress signaling and osmoadaptation in yeasts. Microbiol. Mol. Biol. Rev..

[103] Wang Y., Xu S., Liu Y., Wang J., Wang Y., Huang B. (2018). The two-component system regulators *BcSkn7* and *BcBrrg1* are essential for stress response and virulence in *Botrytis cinerea*. Fungal Genet. Biol..

[104] Brewster J.L., Gustin M.C. (2014). Hog1: 20 years of discovery and impact. Sci. Signal..

