# Supplementary material for: BcHK71 and BcHK67, Two-Component Histidine Kinases, Regulate Conidial Morphogenesis, Glycerol Synthesis, and Virulence in Botrytis cinerea
Source: J Fungi (Basel). 2025 Nov 29;11(12):850. doi: 10.3390/jof11120850 (PMC12733437; doi:10.3390/jof11120850)
Supplement: Supplementary file 1 [file jof-11-00850-s001.zip › Supplementary Material.pdf]

Table S1. Primers used in this study.

Name	Sequence (5′-3′)
BcHK71-innerF	GAGAATGAAGGCCATGTCATTGCT
BcHK71-innerR	ACTCTGCTTGGATCTAACAAGGTC
BcHK67-innerF	GGTACTTGAAGTGGGAGATACGAC
BcHK67-innerR	GGATAAGCAGCAGCTATTATCCAG
BcHK71-UP	GTAGAAGTATTGTCGGCACAGGAG
BcHK71-DN	TCGGCATCACATCTCAAGACCCAT
BcHK67-UP	GACCAACCCTCAAACCCTCAATTC
BcHK67-DN	GACTAACATCCCACATAGTGGCAC
BcHK71UP-F	ACGACGGCCAGTGCCAAGCTTGTCGAGATCTTTCCACAATTTAGA
BcHK71UP-R	CCAGGATCCTCTAGAGTCGACGTTACCAGTTGCAGAACCTTCTTC
BcHK71DN-F	ATTATTATGGAGAAACTCGAGCGAAACCTACGACCCCTCTCACCC
BcHK71DN-R	CCGGGTACCGAGCTCGAATTCGATTTTCCTATTCCTTAGGTTGCC
BcHK67UP-F	GCCAAGCTTGCATGCCTGCAGGTAATAAAGCTAGCTATAAGAGAA
BcHK67UP-R	CCAGGATCCTCTAGAGTCGACGGGTGAGAAAGGGGGACAAATGGT
BcHK67DN-F	CCGGGTACCGAGCTCGAATTCTGCAAAGTTCTACTGTCACCTACT
BcHK67DN-R	ATTATTATGGAGAAACTCGAGTCCTGTCCAGGAAGAAGATTGGTT
HPH-F	TTCGCCCTTCCTCCCTTTATTTCA
HPH-R	GCTTCTGCGGGCGATTTGTGTACG
HPH-UP	ACTGCTACAAGTGGGGCTGATCTG
HPH-DN	CTGGACCGATGGCTGTGTAGAAGT
HK71RTF	CCGTGACGGCTAATGCGAGGAAAGACC
HK71RTR	CCGTGTCGCTCTCAGACTTCGGATACC
HK67RTF	AGTCCTAAGCAAACAACTTCGTAGCGCAGG
HK67RTR	CCTGACACGCCTTCTGGTAGACTTGTGG
UCE-F	ACCGGAGGTGTCTTCTTCCTCGCC
UCE-R	ATGCTTCCGTTGGAGTTGATGTTTGGGT
BcYpd1-F1	ACTCCGGTACCAGCGCCAAAGACAGT
BcYpd1-R1	CTGGTAGACCAACGGACCCATCATCACT
Brrg1-F1	ACTTCCTCGGTCGGATCCGCGAATAATG
Brrg1-R1	GATTACCAGAACCTGTACCCGTGCCGTT
BcSKN7-F1	AGACGAAAGGCACCAGCTCCGAGAAAG
BcSKN7-R1	TCCGACGAGGCCATGCATAACTCCTTC
Bcatf1-F	CGCCGATTCCACCGTGCCAGATAT
Bcatf1-R	GTGTGAGTTGTTGGCCAACGCTCG
BcPtpa-F	CCAGGTCCTTCCTTACTACGCCCA
BcPtpa-R	AGGAAGAAGGCAGTCTCTCAGACG
Bos4-F	GCTCGGAGATTTCACCTCGTGCTG
Bos4-R	CGTGGGAATCTGCAGAAGAACCAG
Bos5-F	ATCCGCAACTAGATGCGATGGGTC
Bos5-R	CCGAATCTGATGCGGGTCGAAGAG
BcartrB-F	ACCGGTGTTACGGTCCCAACTGAG
BcartrB-R	AGCGATGGCGGTGGTAGGGTAATC
BcSak1(Hog1)-F	CGGCACCACCTTTGAGATTA
BcSak1(Hog1)-R	AACCGGTGAGGTTATCTTTGG
BcBot1-F	CGTCAGTGACCCTGACTTCTTCCATGAGG
BcBot1-R	GCTGAGTGCGTTCTGGACAACCTTGA
BcBot2-F	CCAGTCAAGAGATGCAACAGCGGTGG

Table S1. Primers used in this study.

BcBot2-R	GTCGTAAACGTGCTGCGGCACATTG
BcBot3-F	ACAACTCGCATCACTTGTCAGTCTCCCAC
BcBot3-R	ACCAGATCCCTTGAGACCCACGAGTC
BcBot4-F	CATCCTGTGTAGATTCATCGCCGTGTGG
BcBot4-R	AGAGTGGATCCTACCCCGAGAGCTATG
BcBot5-F	ACTACCCAGACCCACAGGGTCTACAC
BcBot5-R	ATGTGCCCATGGCGTCAGTGAGAG

Table S2. Protein sequences used in the phylogenetic analysis of BcHK71 and BcHK67.

No.	Species	Accession Number	Protein Size (aa)	Used in Analysis	Notes / Group
1	*Botrytis cinerea*	(BcHK71 itself)	1,148	BcHK71	Query Sequence, Sclerotiniaceae
2	*Botrytis cinerea*	(BcHK67 itself)	1,244	BcHK67	Query Sequence, Sclerotiniaceae
3	*Ciborinia camelliae*	KAI9651068.1	1075	BcHK71	Sclerotiniaceae Cluster
4	*Monilinia fructicola*	KAG4030056.1	1163	BcHK71	Sclerotiniaceae Cluster
5	*Ciborinia camelliae*	KAI9645299.1	1248	BcHK67	Sclerotiniaceae Cluster
6	*Monilinia fructicola*	KAG4027074.1	1243	BcHK67	Sclerotiniaceae Cluster
7	*Colletotrichum fioriniae*	KAJ0316613.1	1202	BcHK71	Outgroup (Other Ascomycetes)
8	*Diplocarpon rosae*	PBP17289.1	1008	BcHK71	Outgroup (Other Ascomycetes)
9	*Fusarium oxysporum*	KAI7771180.1	1508	BcHK71	Outgroup (Other Ascomycetes)
10	*Lachnellula hyalina*	XP_031005294.1	1043	BcHK71	Outgroup (Other Ascomycetes)
11	*Pyricularia oryzae*	KAH8836968.1	1403	BcHK71	Outgroup (Other Ascomycetes)
12	*Rhynchosporium secalis*	CZT53029.1	1032	BcHK71	Outgroup (Other Ascomycetes)
13	*Saccharomyces cerevisiae*	KAJ1047856.1	622	BcHK71	Outgroup (Yeast)
14	*Cadophora malorum*	KAG4413929.1	1210	BcHK67	Outgroup (Other Ascomycetes)
15	*Colletotrichum fioriniae*	XP_053044864.1	1271	BcHK67	Outgroup (Other Ascomycetes)
16	*Fusarium oxysporum*	KAF5258207.1	1508	BcHK67	Outgroup (Other Ascomycetes)
17	*Lachnellula arida*	TVY16498.1	1214	BcHK67	Outgroup (Other Ascomycetes)
18	*Pyricularia oryzae*	KAI6354880.1	1403	BcHK67	Outgroup (Other Ascomycetes)
19	*Rhynchosporium commune*	CZT09564.1	1196	BcHK67	Outgroup (Other Ascomycetes)
20	*Saccharomyces cerevisiae*	KAF4002908.1	1220	BcHK67	Outgroup (Yeast)

Table S3. Comparison in the changes of gene expression determined by DESeq2 sequencing and qPCR approaches.

Genes name	Δ*BcHK71* Fold change in gene expression determined by DESeq2 sequencing	Δ*BcHK71* Fold change in gene expression determined by qPCR	Regulate	*ΔBcHK67* Fold change in gene expression determined by DESeq2 sequencing	*ΔBcHK67* Fold change in gene expression determined by qPCR	Regulate
BcBot1	*6.95*	*8.71*	up	*6.47*	*60.54*	up
BcBot2	*8.42*	*1.74*	up	*6.91*	*206.97*	up
BcBot3	*6.13*	*17.84*	up	*5.78*	*29.99*	up
BcBot4	*6.86*	*1.55*	up	*3.59*	*3.87*	up
BcBot5	*4.08*	*1.29*	up	*5.81*	*19.56*	up
BcBos4	1.23	3.20	up	1.40	2.75	up
BcBos5	-0.08	0.66	normal	0.16	0.74	normal
BcSak1(Hog 1)	-0.87	0.85	normal	-0.39	0.36	normal
Bcatf1	-0.05	1.36	normal	0.25	0.43	normal
BcPtpa	0.39	1.00	normal	-0.03	0.44	normal

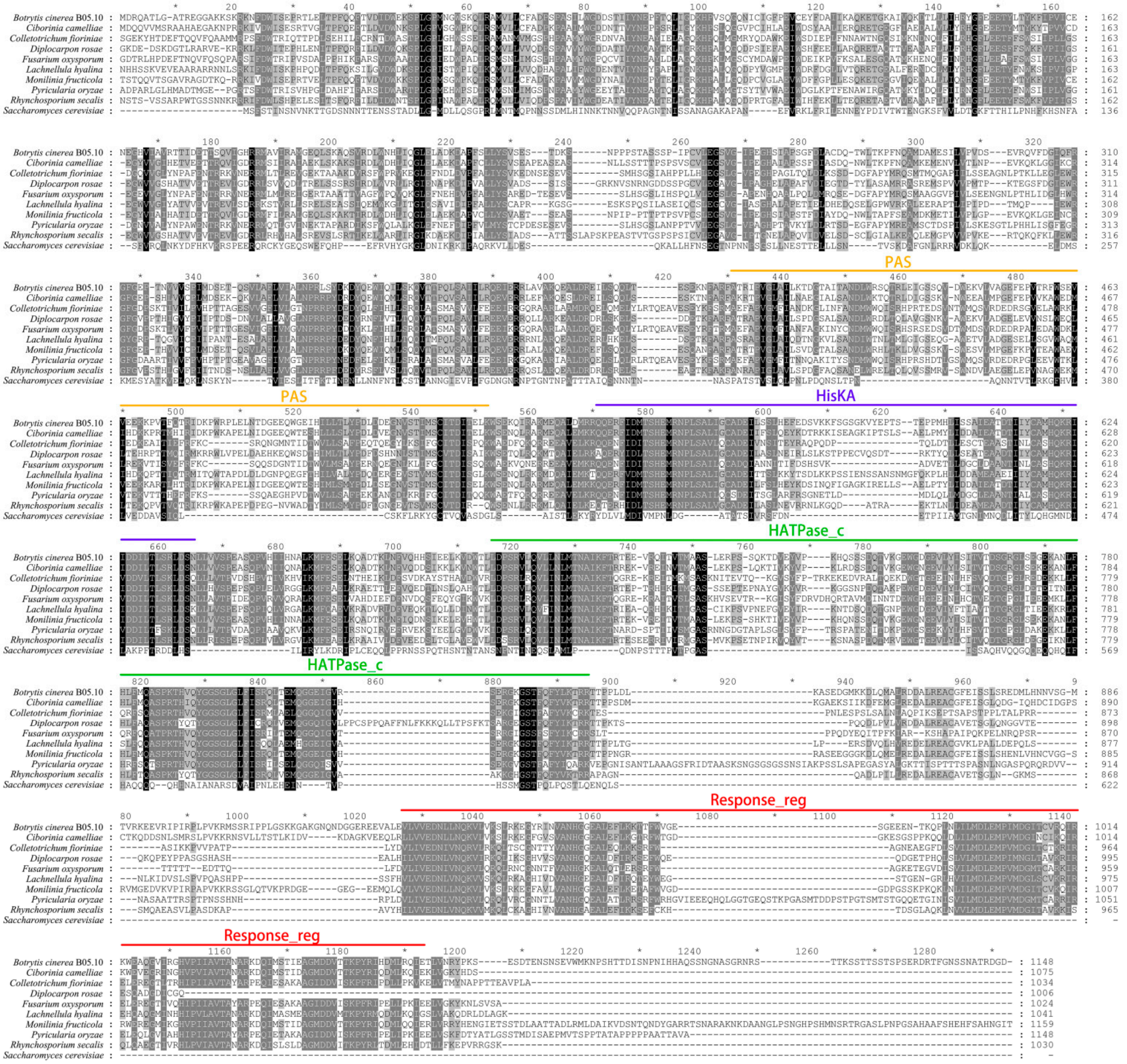

Figure S1. Multiple sequence alignment of BcHK71 and its homologs. Protein sequences were aligned using the Clustal W algorithm. Residues are shaded to indicate conservation: black for identical, dark gray for conserved, and light gray for similar amino acids. The asterisk (*) denotes a position every 10 residues. Species and accession numbers used in the analysis are: *Botrytis cinerea* B05.10 (XP_024553284.1), *Ciborinia camelliae* (KAI9651068.1), *Colletotrichum fioriniae* (KAJ0316613.1), *Diplocarpon rosae* (PBP17289.1), *Fusarium oxysporum* (KAI7771180.1), *Lachnellula hyalina* (XP_031005294.1), *Monilinia fructicola* (KAG4030056.1), *Pyricularia oryzae* (KAH8836968.1), *Rhynchosporium secalis* (CZT53029.1) and *Saccharomyces cerevisiae* (KAJ1047856.1).

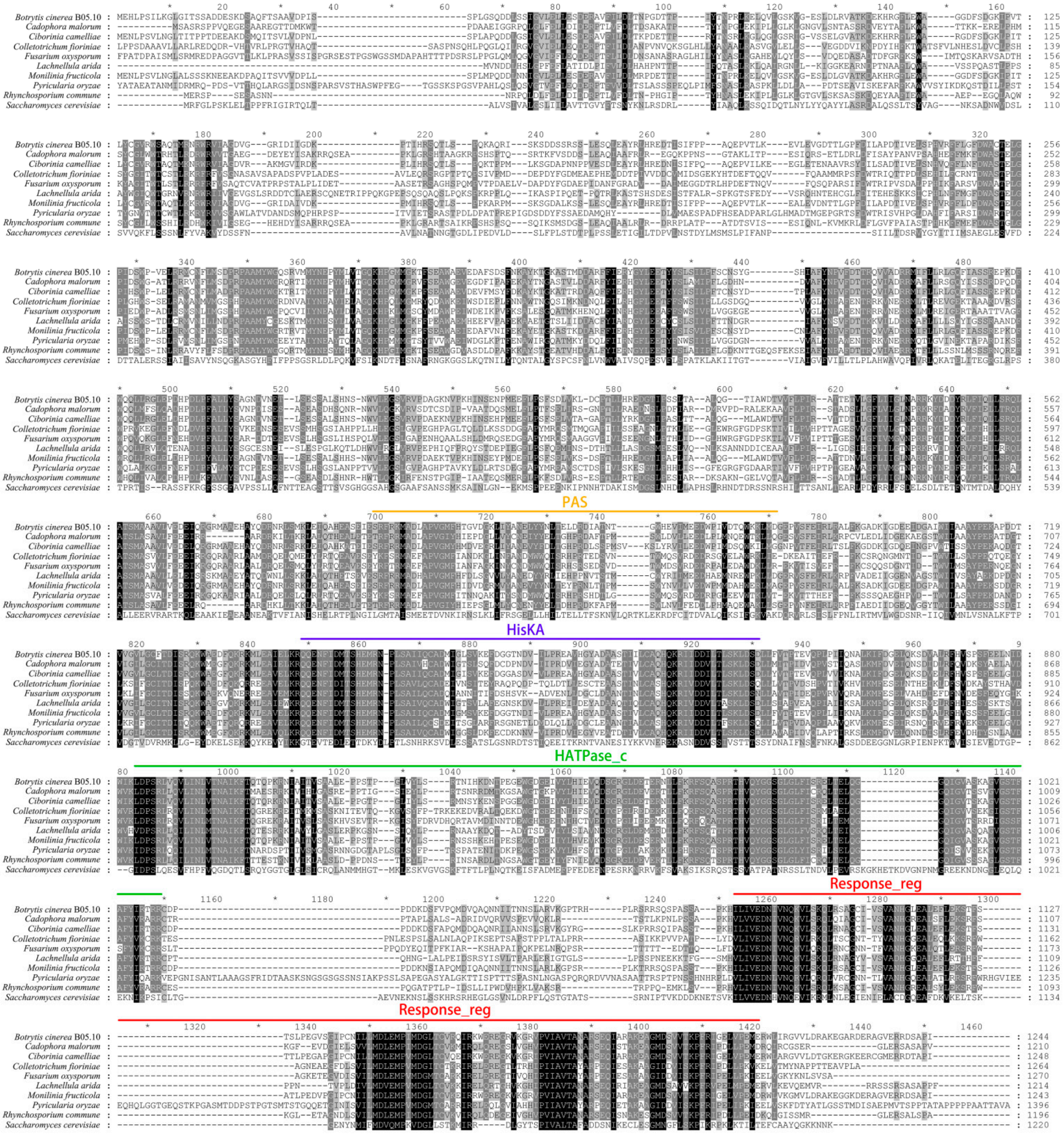

Figure S2. Multiple sequence alignment of BcHK67 and its homologs. Protein sequences were aligned using the Clustal W method. The alignment highlights identical (black), conserved (dark gray), and similar (light gray) residues within the conserved block. An asterisk (*) marks every 10th residue position for reference. The species and corresponding GenBank accession numbers are: *Botrytis cinerea* B05.10 (XP_024545917.1), *Cadophora malorum* (KAG4413929.1), *Ciborinia camelliae* (KAI9645299.1), *Colletotrichum fioriniae* (XP_053044864.1), *Fusarium oxysporum* (KAF5258207.1), *Lachnellula arida* (TVY16498.1), *Monilinia fructicola* (KAG4027074.1), *Pyricularia oryzae* (KAI6354880.1), *Rhynchosporium commune* (CZT09564.1) and *Saccharomyces cerevisiae* (KAF4002908.1).

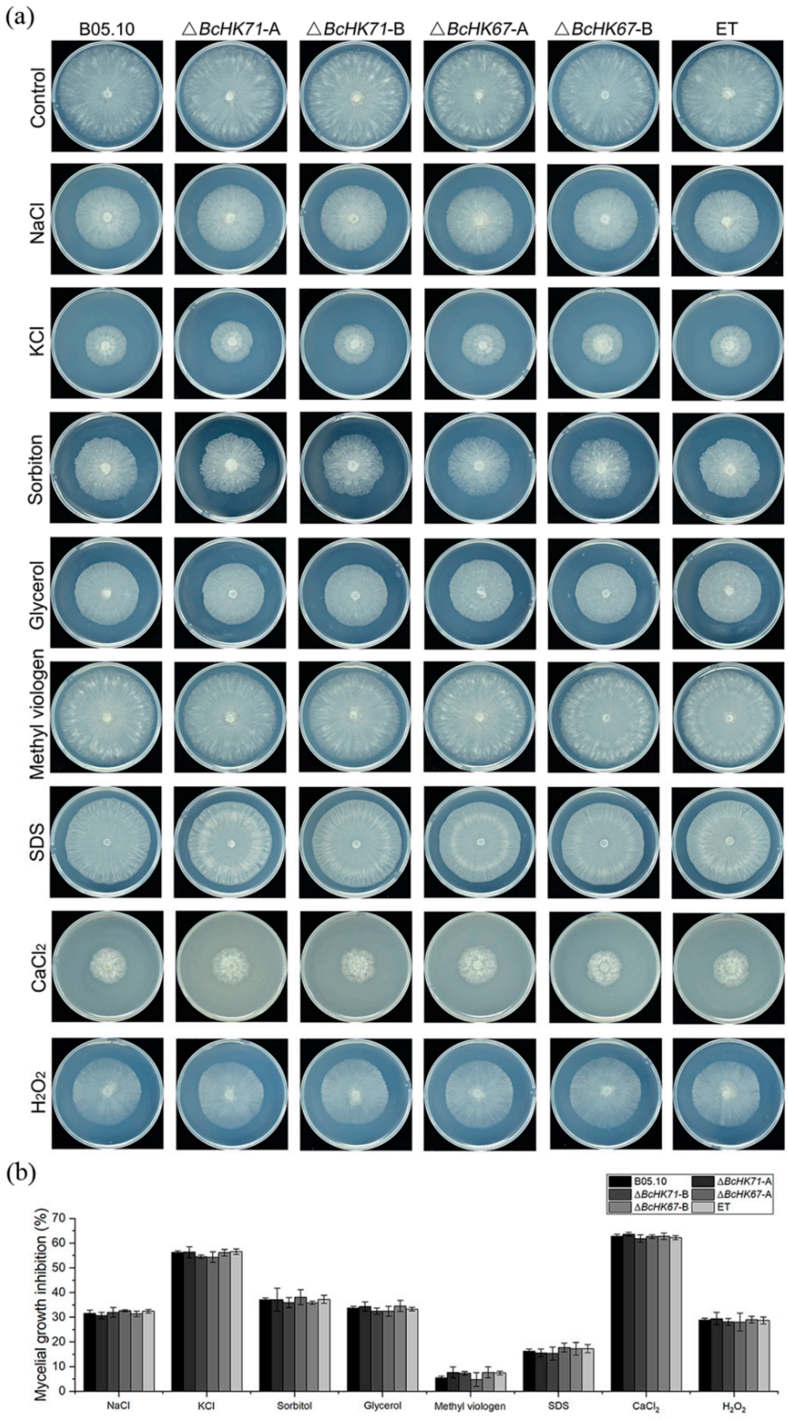

Figure S3. Response of B. cinerea histidine kinase mutants to abiotic stress. (a) Colony morphology of the WT, Δ*BcHK71* and Δ*BcHK67* mutants, and ET strains on CM plates supplemented with various stressors for 3 days. Stressors include osmotic stress(1M NaCl, KCl, sorbitol or glycerol) , a membrane disruptor (0.005% SDS) ; oxidative stress (5mM H_2O_2, 2 mM methyl viologen), and an ionic stressor (0.5 M $CaCl_2$). (b) Relative mycelial growth inhibition under the corresponding stress conditions. Data are presented as mean ± SD (n=3). Significant differences compared to the WT are indicated (ANOVA, Dunnett's test).

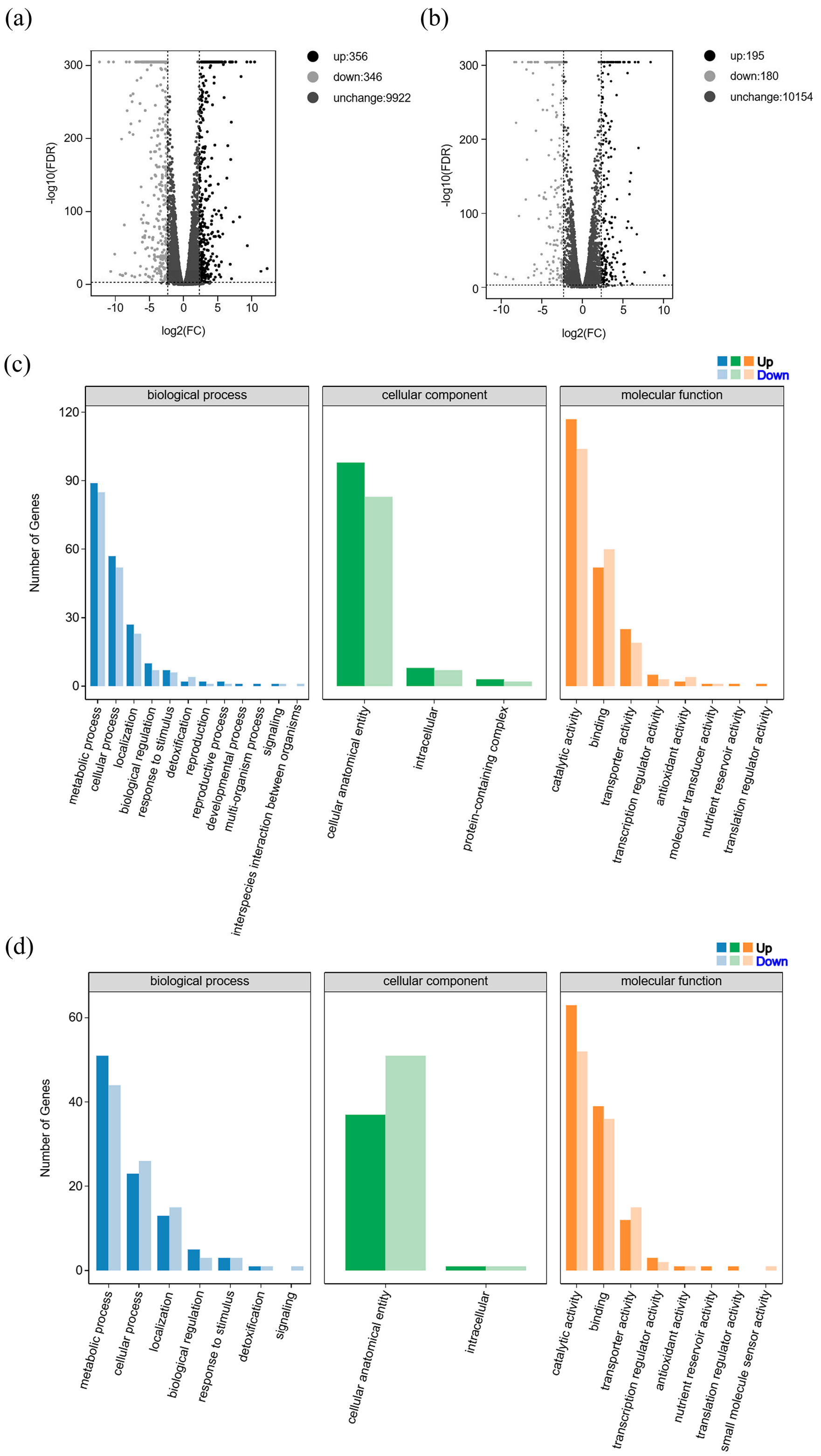

Figure S4. Transcriptomic profiles of Δ*BcHK71* and Δ*BcHK67* mutants. (a, b) Volcano plots of differentially expressed genes (DEGs) identified in the Δ*BcHK71* (a) and Δ*BcHK67* (b) mutants compared to the WT. Dashed lines indicate the thresholds for DEG identification: $|\log_2 FC| \geq 5$ and FDR < 0.001. (c, d) GO functional enrichment analysis of DEGs from the Δ*BcHK71* (c) and Δ*BcHK67* (d) mutants.

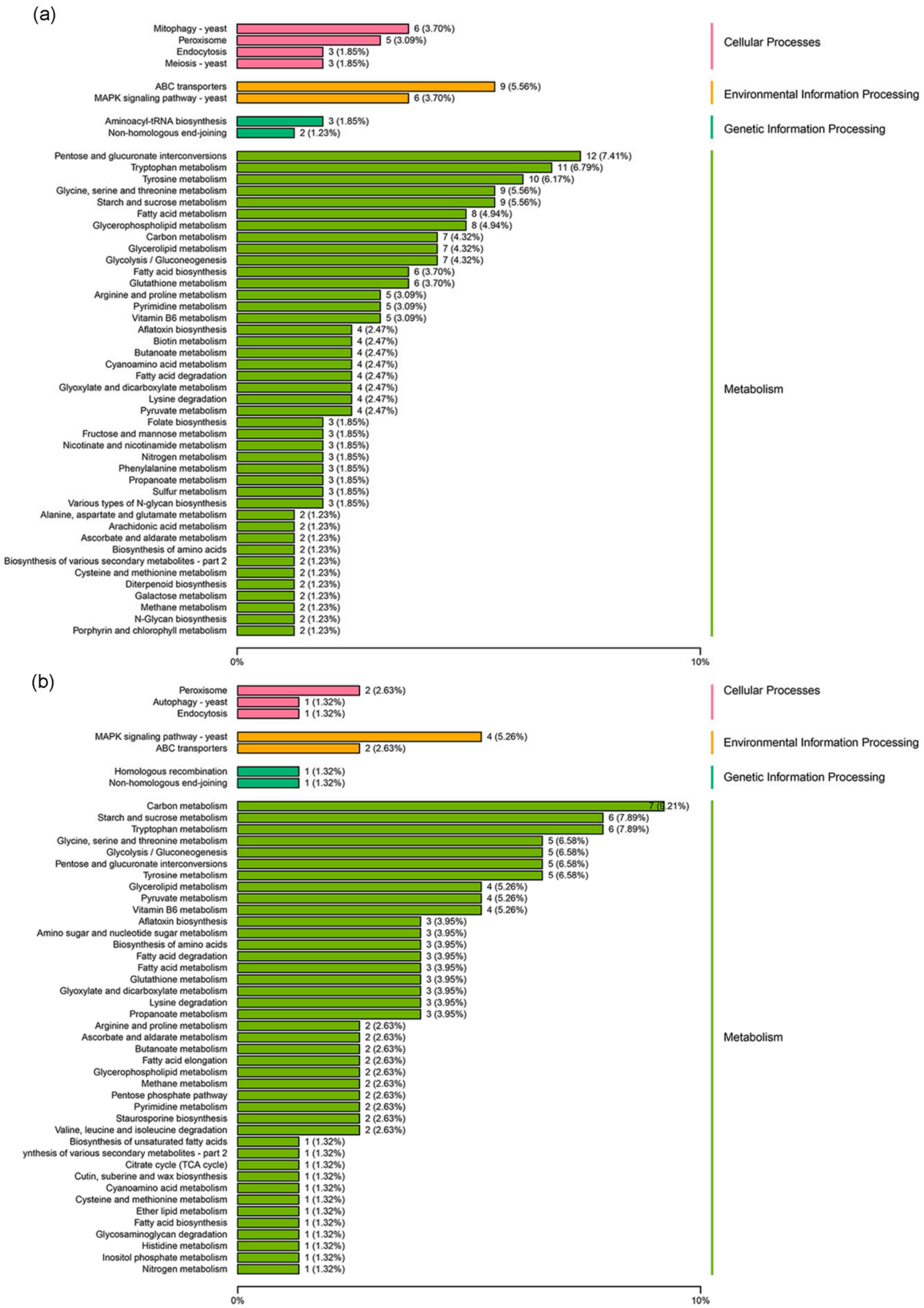

Figure S5. KEGG pathway enrichment analysis of differentially expressed genes (DEGs). (a) Δ*BcHK71* versus WT; (b) Δ*BcHK67* versus WT. DEGs were identified using the thresholds $|\log_2 FC| \geq 5$ and FDR < 0.001.

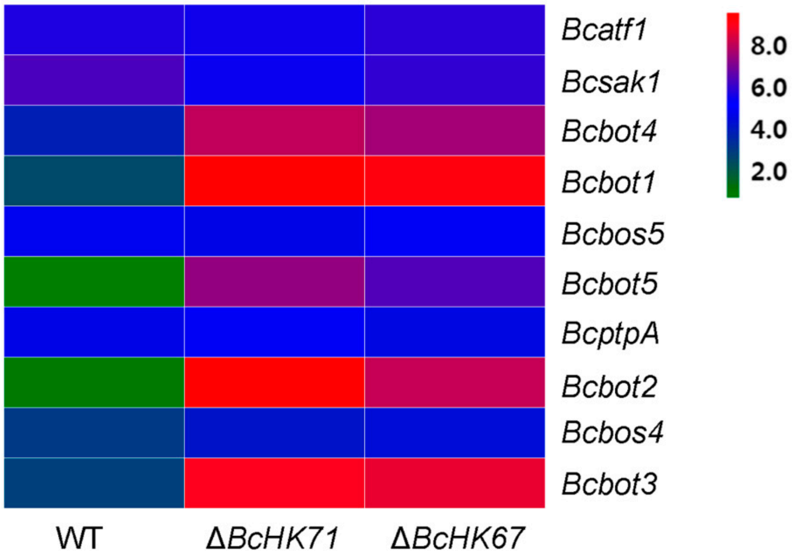

Figure S6. Expression heatmap of selected differentially expressed genes. The heatmap displays normalized expression values for key genes, including botrydial biosynthetic genes (*Bcbot1*-*Bcbot5*), the cutinase gene *Bccaf1*, and core components of the HOG-MAPK pathway (*Bcsak1*, *Bcbos4*, *Bcbos5*, and *BcptpA*).

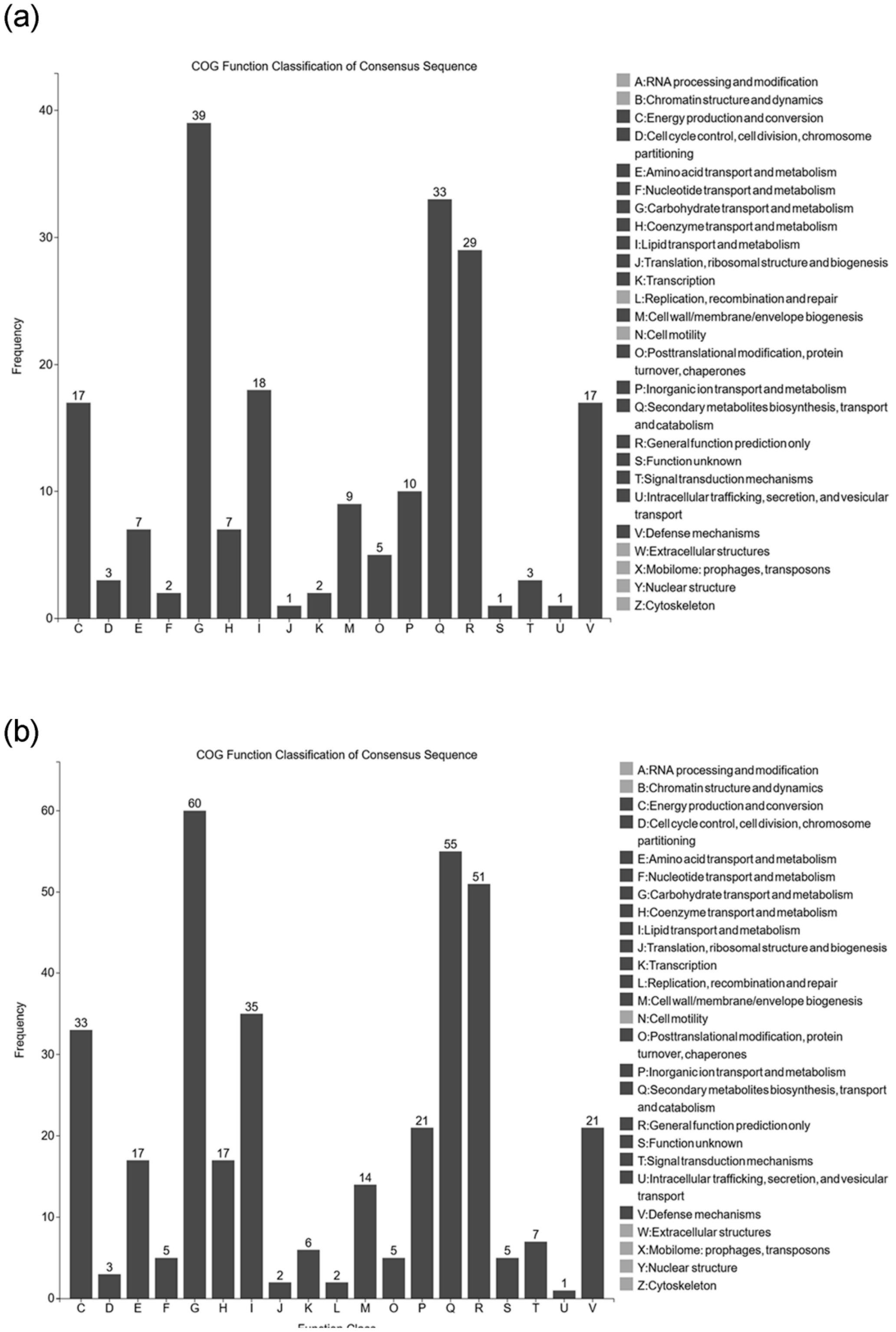

Figure S7. Functional categorization of DEGs based on COG database. (a) COG classification of DEGs from the Δ*BcHK71* mutant versus WT; (b) COG classification of DEGs from the Δ*BcHK67* mutant versus WT. The analysis was performed with a threshold of $|\log_2 FC| \geq 5$ and FDR < 0.001.
